# Exosomal Aβ-Binding Proteins Identified by “In Silico” Analysis Represent Putative Blood-Derived Biomarker Candidates for Alzheimer´s Disease

**DOI:** 10.3390/ijms22083933

**Published:** 2021-04-11

**Authors:** Tânia Soares Martins, Rui Marçalo, Maria Ferreira, Margarida Vaz, Raquel M. Silva, Ilka Martins Rosa, Jonathan Vogelgsang, Jens Wiltfang, Odete A. B. da Cruz e Silva, Ana Gabriela Henriques

**Affiliations:** 1 Neurosciences and Signalling Group, Department of Medical Sciences, Institute of Biomedicine (iBiMED), University of Aveiro (UA), 3810-193 Aveiro, Portugal; martinstania@ua.pt (T.S.M.); ruifilipemarcalo@ua.pt (R.M.); mariaferreira@ua.pt (M.F.); margaridavaz@ua.pt (M.V.); ilkamartins@ua.pt (I.M.R.); Jens.Wiltfang@med.uni-goettingen.de (J.W.); odetecs@ua.pt (O.C.S.); 2 Center for Interdisciplinary Research in Health (CIIS), Faculdade de Medicina Dentária, Universidade Católica Portuguesa, Estrada da Circunvalação, 3504-505 Viseu, Portugal; raquelsilva@ua.pt; 3 Department of Psychiatry and Psychotherapy, University Medical Center Goettingen (UMG), Georg-August University, Von-Siebold-Str. 5, 37075 Goettingen, Germany; jonathan.vogelgsang@med.uni-goettingen.de; 4 Translational Neuroscience Laboratory, McLean Hospital, Harvard Medical School, Belmont, MA 02478, USA; 5 German Center for Neurodegenerative Diseases (DZNE), Von-Siebold-Str. 3a, 37075 Goettingen, Germany

**Keywords:** exosomes, Alzheimer’s disease, Abeta binding proteins, diagnosis, biomarker

## Abstract

The potential of exosomes as biomarker resources for diagnostics and even for therapeutics has intensified research in the field, including in the context of Alzheimer´s disease (AD). The search for disease biomarkers in peripheral biofluids is advancing mainly due to the easy access it offers. In the study presented here, emphasis was given to the bioinformatic identification of putative exosomal candidates for AD. The exosomal proteomes of cerebrospinal fluid (CSF), serum and plasma, were obtained from three databases (ExoCarta, EVpedia and Vesiclepedia), and complemented with additional exosomal proteins already associated with AD but not found in the databases. The final biofluids’ proteomes were submitted to gene ontology (GO) enrichment analysis and the exosomal Aβ-binding proteins that can constitute putative candidates were identified. Among these candidates, gelsolin, a protein known to be involved in inhibiting Abeta fibril formation, was identified, and it was tested in human samples. The levels of this Aβ-binding protein, with anti-amyloidogenic properties, were assessed in serum-derived exosomes isolated from controls and individuals with dementia, including AD cases, and revealed altered expression patterns. Identification of potential peripheral biomarker candidates for AD may be useful, not only for early disease diagnosis but also in drug trials and to monitor disease progression, allowing for a timely therapeutic intervention, which will positively impact the patient’s quality of life.

## 1. Introduction

Alzheimer’s disease (AD) is the most common, irreversible and progressive neurodegenerative disorder worldwide. Its progression leads to alterations in cognitive functions, such as memory and orientation that will interfere with daily life activities and impact the patient’s quality of life. The formation of extracellular senile plaques, composed of deposits of the amyloid beta peptide (Aβ), and intracellular neurofibrillary tangles, as a result of Tau hyperphosphorylation, are two main disease histopathological hallmarks [1].

At the disease diagnostic level, a wide range of clinical cognitive tests are employed to evaluate cognitive deficits and also imaging tools, which have improved considerably with emerging techniques like positron emission topography (PET) [2,3]. In addition, the detection of the biomarker triplet, Aβ42, T-Tau and p-Tau-181 in cerebrospinal fluid (CSF) [4,5], is currently the gold standard for neurochemical-based diagnosis, employed to discriminate AD from other dementia subtypes. The clinical value of this diagnosis is unquestionable; however, a limitation arises due to the invasive procedure for CSF collection. Therefore, research trends have focused on identifying novel biomarker candidates in more accessible and peripheral biofluids such as blood [6].

Exosomes, a subclass of extracellular vesicles (EVs) with a multivesicular endosomal origin, ranging from 30 to 150 nm, are formed by a lipid bilayer membrane enclosing a free-cytosol organelle, composed by several macromolecules (also called luminal cargo). Its cargo can comprise proteins, RNA, DNA and lipids [7], which arouse interest in using these nanovesicles as novel putative diagnostic resources for several diseases, including, more recently, AD [8,9,10]. These nanovesicles are secreted by all cell types in the human organism, including neurons, and can carry disease molecular profiles. In fact, exosomes can carry specific pathogenic content linked to AD. In the context of AD, exosomes can transport both Aβ and Tau and act as active players in the spread of these pathogenic species, triggering abnormal/neurodegenerative processes that contribute to disease progression [11,12,13]. Likewise, a protective role has been attributed to these types of EVs, since exosomes can also carry Aβ degrading enzymes; and exosome infusions have proven to be useful in the clearance of Aβ deposits [14,15,16,17,18]. Several other exosomal candidates have been identified as putative biomarkers for AD diagnosis. These include, not only Aβ and Tau species, but also inflammation-related mediators, like complement effector and regulatory proteins, synaptic proteins, growth factors and insulin signaling receptors [10,19,20,21,22]. Since Aβ is transported in exosomes and their levels are altered with AD in the majority of the studies [8], this encourages the search for Aβ-binding proteins in exosomes. This group of proteins, in essence the Aβ interactome, can interact with monomeric, oligomeric and fibrillar forms of Aβ and modulate its clearance and degradation, promote amyloid aggregation and influence its transport [23].

Hence, the aim of this study was to address the exosomal proteome from three distinct biofluids, namely, serum, plasma and CSF, for comparative purposes, and to identify Aβ-binding proteins within these proteomes that may constitute putative AD biomarker candidates.

## 2. Results and Discussion

### 2.1. Exosomal Proteomes Gene Ontology (GO) Analysis

**Figure 1:**
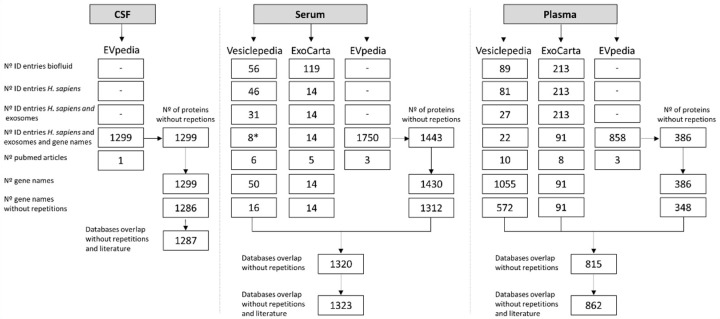
Exosomal proteomes obtained for the distinct biofluids. Steps implemented to obtain the final proteomes using the three databases (Vesiclepedia, ExoCarta, EVpedia) for cerebrospinal fluid (CSF), serum and plasma. A set of exclusion filters was used in this order: number of entries for the biofluid, number of ID entries for *Homo sapiens*, number of ID entries for *H. sapiens* and exosomes, number ID entries for *H. sapiens* and exosomal gene names, number of corresponding PubMed articles, number of gene names and number of gene names without repetitions. Subsequently, the lists obtained for the three databases were overlapped, the repetitions deleted and, the gene names corresponding to the exosomal proteins identified in the literature for CSF, serum or plasma were included in the final lists. The EVpedia’s obtained Uniprot IDs were converted to gene names. In the case of CSF, only EVpedia had information available. * One study was excluded since it was related to urine exosomal proteome and not to serum.

Exosomal proteomes derived from CSF, serum and plasma were obtained by accessing three online databases (ExoCarta, Vesiclepedia and EVpedia). For each database, the proteins found in exosomes were collected and a list of proteins/corresponding genes present was produced for each biofluid. The final biofluid proteome lists also include proteins described in the literature in AD-related studies and not yet entered in the databases (Supplementary Table S1). The final number of exosomal proteins in the biofluids lists was 1287 for CSF, 1323 for serum and 862 for plasma (Figure 1).

**Figure 2:**
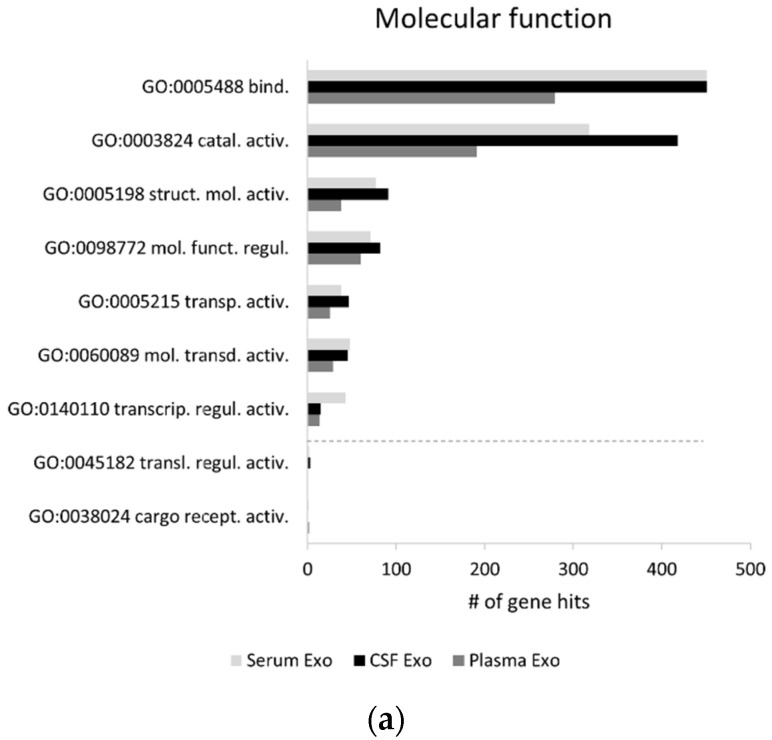

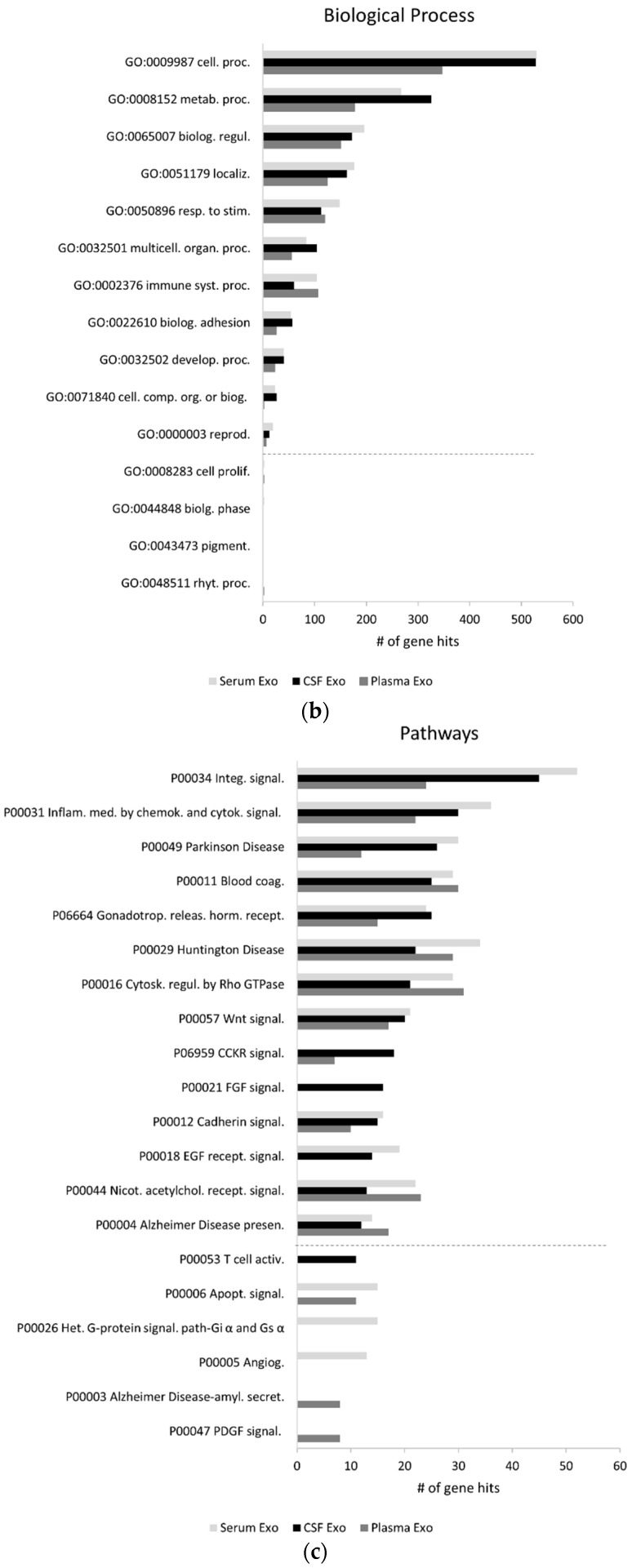
Characterization of the exosomal proteome from distinct biofluids. Exosomal proteomes associated molecular functions (**a**), biological processes (**b**) and pathways (**c**) analysis. Gene ontology (GO) analysis was performed for gene lists corresponding to the exosomal proteomes from serum, plasma and CSF, through the Panther classification system. Exclusive GO terms for exosomes isolated from only one biofluid or shared only by exosomes from two biofluids were represented below the dashed lines. Abbreviations of molecular function GOs terms: GO:0005488 binding; GO:0003824 catalytic activity; GO:0005198 structural molecule activity; GO:0098772 molecular function regulator; GO:0005215 transporter activity; GO:0060089 molecular transducer activity; GO:0140110 transcription regulator activity; GO:0045182 translation regulator activity; GO:0038024 cargo receptor activity. Abbreviations of biological process GOs terms: GO:0009987 cellular process; GO:0008152 metabolic process; GO:0065007 biological regulation; GO:0051179 localization; GO:0050896 response to stimulus; GO:0032501 multicellular organismal process; GO:0002376 immune system process; GO:0022610 biological adhesion; GO:0032502 developmental process; GO:0071840 cellular component organization or biogenesis; GO:0000003 reproduction; GO:0008283 cell proliferation; GO:0044848 biological phase; GO:0043473 pigmentation; GO:0048511 rhythmic process. Abbreviations of pathways GOs terms: P00034 integrin signaling pathway; P00031 inflammation mediated by chemokine and cytokine signaling pathway; P00049 Parkinson disease; P00011 blood coagulation; P06664 gonadotropin-releasing hormone receptor pathway; P00029 Huntington disease; P00016 cytoskeletal regulation by Rho GTPase; P00057 Wnt signaling pathway; P06959 CCKR signaling map; P00021 FGF signaling pathway; P00012 cadherin signaling pathway; P00018 EGF receptor signaling pathway; P00044 nicotinic acetylcholine receptor signaling pathway; P00004 Alzheimer’s disease-presenilin pathway; P00053 T cell activation; P00006 apoptosis signaling pathway; P00026 heterotrimeric G-protein signaling pathway-Gi alpha and Gs alpha mediated pathway; P00005 angiogenesis; P00003 Alzheimer’s disease-amyloid secretase pathway; P00047 PDGF signaling pathway. Exo, Exosomes.

The characterization and comparison of the exosomal proteomes is relevant since it can give important clues into the main processes and pathways to which the distinct biofluid proteomes are most associated, allowing one to potentially explore new disease biomarker candidates. GO enrichment analysis was thus performed, at the level of the molecular function and biological process; and the major pathways linked to these proteomes were addressed. A global GO analysis for these biofluids’ exosomal proteomes was carried out using the Panther classification system online tool (Figure 2). At the molecular function level, the top five functions identified were the same for all exosomal proteomes and included “binding”, “catalytic activity”, “structural molecule activity”, “molecular function regulator” and “transporter activity” (Figure 2a).

Regarding the top 5 biological processes found for these three proteomes, similar processes were likewise identified, namely “cellular process”, “metabolic process”, “biological regulation”, “localization” and “response to stimulus” (Figure 2b). However, the “rhythmic process” was exclusive to plasma exosomes and “pigmentation” and “biological phase” were biological processes exclusive to serum exosomes. This is potentially relevant when directing studies aimed at addressing particular processes in distinct biofluids.

Major differences arise when addressing the top exosomal proteomes-associated pathways (Figure 2c). For CSF, the top five pathways linked to the exosomal proteome were “integrin signaling pathway”, “inflammation mediated by the chemokine and cytokine signaling pathway”, “Parkinson disease”, “blood coagulation” and “gonadotropin-releasing hormone receptor pathway”. Similar to CSF, the serum-derived exosomal proteome also associated with the “integrin signaling pathway”, “inflammation mediated by chemokine and cytokine signaling pathway”, “blood coagulation” and “Huntington disease”, but it appeared with an increased number of hits related to the “Parkinson disease” pathway.

Plasma exosomal proteome was the most distinct, being mainly associated with “cytoskeletal regulation by Rho GTPase”, “blood coagulation”, followed by “Huntington disease”, “integrin signaling pathway” and “nicotinic acetylcholine receptor signaling pathway”. In addition, if the top 15 pathways were considered, “T cell activation” and “FGF signaling pathway” appeared as being exclusive for the CSF exosome proteome, while “Alzheimer disease-amyloid secretase pathway” and “Platelet-derived growth factor (PDGF) signaling pathway” appeared as pathways exclusive to the plasma-derived proteome. Likewise, exclusive serum exosomal pathways were found, namely “angiogenesis” and “heterotrimeric G-protein signaling pathway-Gi alpha and Gs alpha mediated pathway”.

In general terms, the main molecular function and biological process were similar for the three biofluids’ exosomal proteomes, however, major differences arise for the pathways associated to the distinct biofluid proteomes. The data available suggest that perhaps plasma can be an ideal biofluid for biomarker studies addressing AD-related candidates, since despite being the smallest proteome, plasma exosomal proteome was associated with two AD processing pathways. However, one cannot exclude that differences in these biofluid proteomes analysis, may be due to the fact that some proteins were still not identified in the distinct biofluids to date or included in databases.

Nonetheless, taken together, data suggest that although the most abundant functions and processes are similar, variances, in particular at the pathway’s levels, can support the use of distinct biofluids for different downstream applications, depending on the research goal.

### 2.2. GO Analysis of the Biofluids Shared Exosomal Proteome 

Cerebrospinal fluid arises as a major source of biomarkers for AD since it represents a route of communication from the brain to the rest of the body. Indeed, gold standard biomarker candidates have already been identified in this biofluid, however, the invasiveness of the CSF collection procedure limits its general use. Hence, in this study the shared proteome of the three biofluids was identified as this would represent a source of putative peripheral disease candidates.

**Figure 3:**
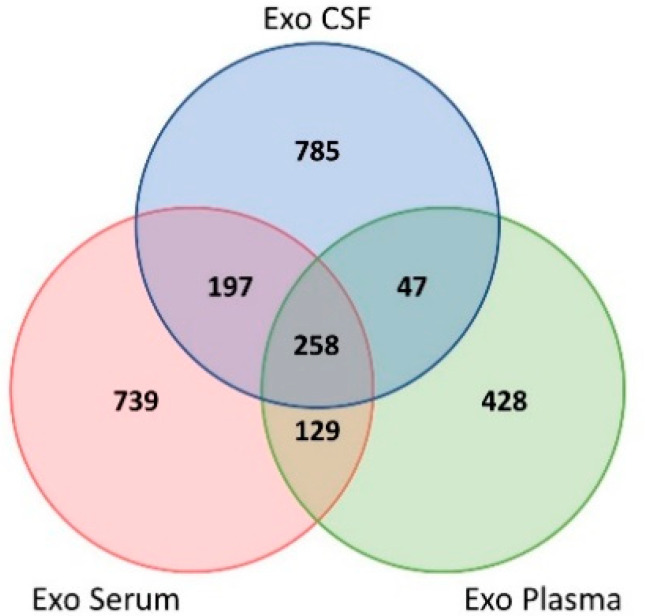
Overlap of the distinct biofluids exosomal proteomes. Venn diagram overlapping serum, plasma and CSF-derived exosomal proteomes. Abbreviations: Exo, exosomes; CSF, cerebrospinal fluid.

To address the shared biofluids’ proteome, the protein lists were compared by means of a Venn diagram (Figure 3). A total of 258 proteins were identified in exosomes from all three biofluids (CSF, plasma, serum). In addition, 197 proteins were shared by both CSF and serum, whereas 47 proteins were shared by both CSF and plasma.

**Figure 4:**
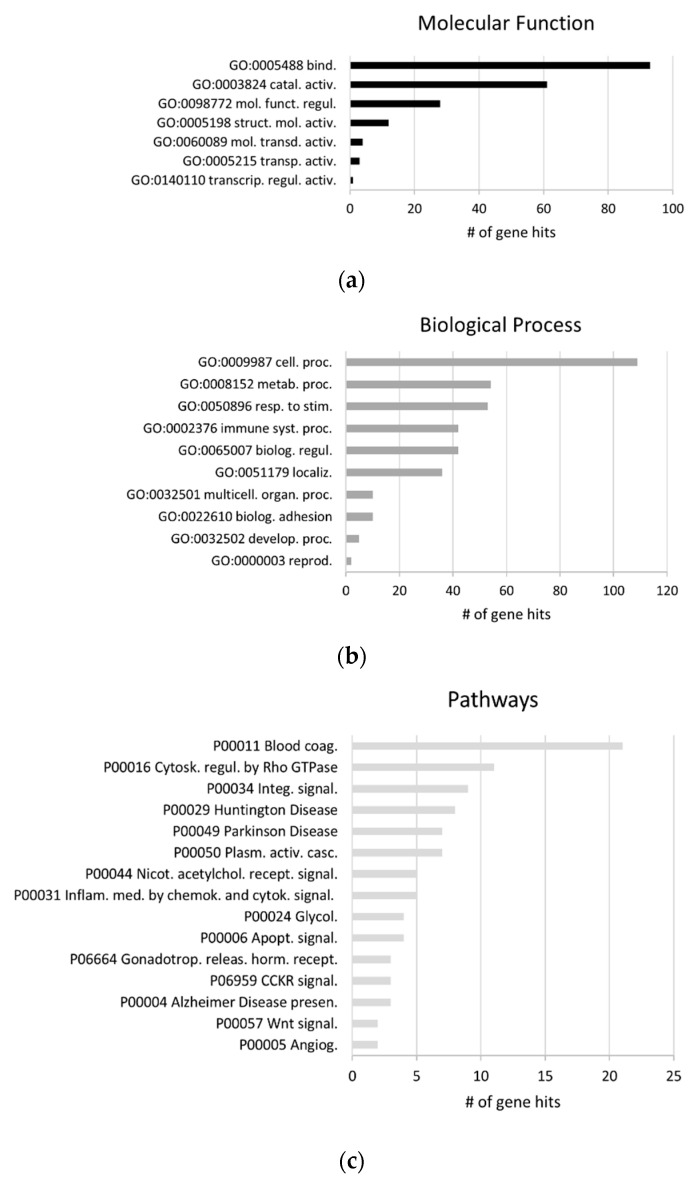
GO analysis of the shared exosomal proteome. Shared exosomal proteome associated molecular functions (**a**), biological processes (**b**) and pathways (**c**). GO analysis was performed for gene lists corresponding to the exosomal proteomes from serum, plasma and CSF, through the Panther classification system. Abbreviations of molecular function GOs terms: GO:0005488 binding; GO:0003824 catalytic activity; GO:0098772 molecular function regulator; GO:0005198 structural molecule activity; GO:0060089 molecular transducer activity; GO:0005215 transporter activity; GO:0140110 transcription regulator activity. Abbreviations of biological process GOs terms: GO:0009987 cellular process; GO:0008152 metabolic process; GO:0050896 response to stimulus; GO:0002376 immune system process; GO:0065007 biological regulation; GO:0051179 localization; GO:0032501 multicellular organismal process; GO:0022610 biological adhesion; GO:0032502 developmental process; GO:0000003 reproduction. Abbreviations of pathways GOs terms: P00011 blood coagulation; P00016 cytoskeletal regulation by Rho GTPase; P00034 integrin signaling pathway; P00029 Huntington disease; P00049 Parkinson disease; P00050 plasminogen activating cascade; P00044 nicotinic acetylcholine receptor signaling pathway; P00031 inflammation mediated by chemokine and cytokine signaling pathway; P00024 glycolysis; P00006 apoptosis signaling pathway; P06664 gonadotropin-releasing hormone receptor pathway; P06959 Cholecystokinin receptor (CCKR) signaling map; P00004 Alzheimer’s disease-presenilin pathway; P00057 Wnt signaling pathway; P00005 angiogenesis.

GO analysis for the three biofluids shared exosomal proteome was carried out (Figure 4). Comparatively to the global proteomes, differences were found in the top five molecular functions, “molecular transducer activity” arises at the top five. The most relevant biological processes associated to these proteins were “cellular process”, “metabolic process”, “response to stimulus”, “biological regulation” and “immune system process”. The latter process arises in the top five, for the shared proteome, in comparison to the general proteomes.

Differences were also evident for the top five pathways, which include “blood coagulation”, “cytoskeleton regulation by Rho GTPase”, “integrin signaling pathway”, “Huntington disease”, and “Parkinson disease”. Indeed, several pathways related with distinct brain disorders, including AD, were evident in this shared proteome.

### 2.3. Potential Blood-Derived Exosomal Candidates for AD 

Considering the interest in identifying putative biomarker candidates for AD in peripheral biofluids, a literature search was performed to identify Aβ-binding proteins in the group of the 258 exosomal proteins that were shared by the three biofluids. A total of 42 Aβ-binding proteins were identified and alterations reported for these protein levels in either biofluids, brain or both, were annotated (Supplementary Table S1 and Table 1).

Important key players in AD pathology were found in this shared exosomal proteome. Among the Aβ-binding proteins is apolipoprotein E (APOE) that can interact and form complexes with Aβ [123,124,125], being that the ApoE4 allele was reported to promote amyloidogenesis [126]. ApoE is also involved in Aβ clearance across the blood-brain barrier and even some isoforms of APOE interact with Tau protein [127,128].

Both alpha-1-antichymotrypsin and alpha-1-antitrypsin are serine protease inhibitors found in senile plaques and neurofibrillary tangles [129], although with opposite roles in AD: alpha-1-antichymotrypsin associates with Aβ, promoting the formation of fibrils and it can also induce tau hyperphosphorylation in neurons [130,131,132] whereas alpha-1-antitrypsin exerts anti-inflammatory properties and, thus, a protective role by mediating the microglial response [133].

Alpha-2-macroglobulin (A2M), clusterin (Clu, also known as ApoJ), prolow-density lipoprotein receptor-related protein 1 (LRP1) and transthyretin (TTR) are other proteins found, that could prevent the misfolding and aggregation of soluble Aβ and/or promoting Aβ clearance. The alpha-2-macroglobulin promotes a protease-mediated Aβ-degradation and acts as a chaperone [134] whereas ApoJ and LRP1 are involved in the Aβ transport across the blood-brain barrier [135,136]. LRP1 can bind to both free Aβ, ApoE/Aβ complexes or alpha-2-macroglobulin/Aβ complexes [137].

Transthyretin is another protective element since it mediates Aβ transport across the blood-brain barrier and can proteolytically cleave Aβ, inhibiting Aβ fibrils formation [138,139].

Complement components were also evident in the proteome lists; among them C1QA subcomponent subunits, C1S, C3, C4B, CFH and CFI. In AD, the complement system is overexpressed in the brain as a response to Aβ stimulation [140]. The group of heat shock proteins represented here by the heat shock protein beta-1 (HSPB1) plays an important role in AD as chaperones due to their ability to refold proteins, acting as anti-amyloidogenic agents.

Another Abeta-binding protein found was gelsolin (GSN), which is a multifunctional actin-binding protein, involved in processes such as cytoskeletal remodeling and cell motility, and it is also considered an amateur chaperone. Several studies reported that gelsolin can prevent Aβ aggregation, exerting neuroprotective roles [141]. This protective role of gelsolin has been the focus of several studies that employed gelsolin infusions or performed its transgene expression and observed a reduction in amyloid pathology [142,143]. GSN exists in two forms, a cytoplasmic form (intracellular) and, as a secreted form in CSF or blood. These differ in length, the plasma secreted form is longer than the intracellular form, and in the disulfide structure [144]. GSN is an abundant protein in blood, present at concentrations around 150–300 µg/mL [141,145] and the levels in plasma correlated with disease progression, assessed by Mini-Mental State Examination (MMSE) [71]. The intracellular form of GSN regulates the actin assembly, and it has an anti-apoptotic and tumor suppressive role [141]. Interestingly, previous work by the group demonstrated that Aβ peptides can form complexes with both gelsolin and laminin, another Aβ-binding protein with neuroprotective impacts, and reverse the peptide intracellular effects. In addition, cell treatment with Aβ decreased the levels of GSN suggesting a potential mechanism to decrease its clearance and enhance neurodegeneration [146]. Taken together, the data prompted an assessment of the levels of gelsolin in serum-derived exosomes using two distinct study groups, one from the pcb-cohort, established at the University of Aveiro (UA), and another from the University Medical Center Goettingen (UMG) cohort. Demographics and clinical data of the UA- and the UMG-study groups is presented in Supplementary Tables S2 and S3, respectively. No differences were found for the mean age, and years of literacy in both groups. Regarding MMSE scores, differences were only found between controls and the UA-dementia group (clinical dementia rating (CDR) ≥ 1 and MMSE+). For the UMG-AD group, CSF biomarker measurements reflected cognitive alterations and, as expected, the CSF Aβ1-42/1-40 ratio was decreased in AD cases while total-Tau and P-Tau levels were increased.

### 2.4. Gelsolin as Putative Exosomal Biomarker Candidate 

Extracellular vesicles compatible with exosome like-characteristics were isolated and characterized as previously described [9,147]. Particle concentration and mode size (Supplementary Figure S1) were assessed by nanoparticle tracking analysis (NTA) revealing no significant differences between controls and individuals with dementia. The nature of the EVs samples was further assessed by Western blot analysis for exosomal markers as TSG101 and HSP70. As expected, these were detected in the EVs preparations, while no signal was detected for calnexin.

**Figure 5:**
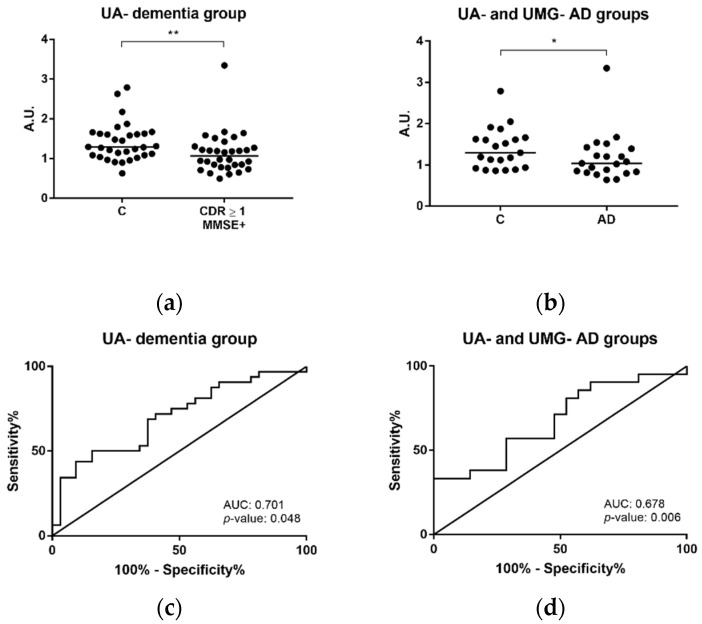
Gelsolin levels in dementia and AD determined by Western blot analysis. Immunoblot analysis of gelsolin in serum-derived exosomes from controls (CDR = 0 and MMSE–) and individuals with dementia (CDR ≥ 1 and MMSE+) from University of Aveiro (UA)-dementia group (**a**), including AD clinically diagnosed cases and controls (UA- and University Medical Center Goettingen (UMG)-groups) (**b**). ROC curves were obtained for the control and dementia group (UA-dementia group) (**c**) and controls and AD cases from UA and UMG (**d**). Each point represents the densitometry ratio obtained for each individual and the solid horizontal line shows median. Abbreviations: AD, Alzheimer’s disease; A.U., arbitrary units; AUC, area under the curve; C, controls; CDR, clinical dementia rating; MMSE, Mini-Mental State Examination; ROC, receiver operating curve. ** *p* ≤ 0.01, * *p* ≤ 0.05.

GSN levels were first evaluated by Western blot analysis, in serum-derived EVs of controls and individuals with dementia, including clinical diagnosed AD cases from the pcb-cohort (Figure 5 and Supplementary Figure S2). The antibody used recognizes both the intracellular and secreted GSN forms. Gelsolin levels were significantly decreased in serum-derived exosomes of individuals with dementia (CDR ≥ 1 and MMSE+) when compared with controls (CDR = 0 and MMSE–) (Figure 5a, *p*-value = 0.005). The same pattern was observed for controls and AD cases of both UA- and UMG- groups (*n* = 21 controls and *n* = 21 AD) (Figure 5b, *p*-value = 0.049). Receiver operating curves (ROC curves) were calculated and the area under the curve (AUC) was approximately 0.7, for both controls and individuals with dementia from the UA–dementia group (Figure 5c) and controls versus AD cases (Figure 5d).

**Figure 6:**
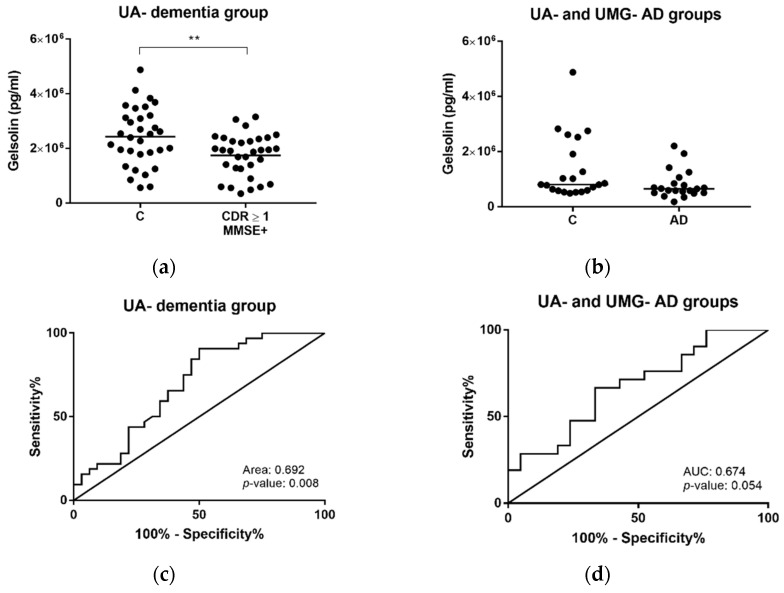
Gelsolin concentrations in dementia and AD cases determined by ELISA. Gelsolin levels were assessed through ELISA assays in serum-derived exosomes from controls (CDR = 0 and MMSE–) and individuals with dementia (CDR ≥ 1 and MMSE+) from UA-dementia group (**a**), and from AD clinically diagnosed cases and controls from UA- and UMG-groups (**b**). ROC curves were obtained for the control and dementia group (UA-dementia group) (**c**) and controls and AD cases from UA- and UMG-groups (**d**). Each point represents the gelsolin mean concentration obtained for each individual and the solid horizontal line shows median of the groups. Abbreviations: AD, Alzheimer’s disease; A.U., arbitrary units; AUC, area under the curve; C, controls; CDR, clinical dementia rating; ELISA, enzyme-linked immunosorbent assay, MMSE, Mini-Mental State Examination; ROC, receiver operating curve. ** *p* ≤ 0.01.

Further, results obtained by Western blot analysis were validated by a complementary approach, using an enzyme-linked immunosorbent assay (ELISA)-based assay for detection of human GSN levels. Consistently, a significant decrease in the median levels of GSN was likewise observed for serum-derived exosomes in the dementia group (CDR ≥ 1 and MMSE+) when compared with controls (CDR = 0 and MMSE+) (*p*-value = 0.008) (Figure 6a). In the case of controls and ADs from both UA- and UMG-groups, the same pattern of decrease was exhibited for AD cases, although without significant differences (*p*-value = 0.055) (Figure 6b). ROC curves were calculated and the AUC obtained was again approximately 0.7 when comparing controls and CDR ≥ 1 and MMSE+ individuals from the UA-dementia group (Figure 6c); and controls and AD cases from UA- and UMG-groups (Figure 6d).

In summary, GSN levels appear to hold potential as a biomarker candidate for dementia/AD. Of note, other Aβ-binding protein candidates identified (laminin and clusterin) can represent likewise interesting targets for AD, that can be tested in future studies. In fact, the use of a combination of blood-derived exosomal biomarkers could undoubtedly increase the sensitivity and specificity of the diagnosis. To validate gelsolin´s potential, also regarding differential diagnosis, its levels should be assessed in a higher number of individuals, with distinct dementia subtypes, or even including other pathologies where GSN levels might also be altered [141,145]. Nonetheless, the results herein presented are in accordance with the reduced levels of plasma and CSF gelsolin and their correlation to the disease progression, already reported in the literature [71,72,73]. To our knowledge, this is the first time that GSN levels were assessed in serum-derived EVs, in the context of AD. GSN has been signaled as an antiamyloidogenic protein in AD, since this protein has the ability to either bind Aβ promoting its clearance, preventing its fibrillization or both. Hence, it is not surprising that if gelsolin is counteracting disease progression in the brain, its levels can be decreased to some degree in blood-derived exosomes; an aspect that deserves further clarification. It would be interesting to simultaneously compare the diagnostic sensitivity and specificity of GSN in serum/plasma and in exosomes-derived from serum/plasma. In addition, as exosomes’ cargos can reflect disease stages it is not surprising that exosomal gelsolin levels in the periphery could change during dementia progression.

## 3. Materials and Methods

### 3.1. Exosomal Proteomes Construction 

Exosomal proteomes for the three biofluids (CSF, serum and plasma) were obtained from three online databases: EXOCARTA, Vesiclepedia and EVpedia (all accessed on 12 January 2020). All databases comprise protein, lipids and nucleic acids data and are all manually curated. EXOCARTA was the first of these databases to be launch and includes data only from exosomes (http://www.exocarta.org/) [148,149,150,151]. Vesiclepedia was launched later and, now, it gathers information from all classes of extracellular vesicles among them exosomes, microparticles and microvesicles (http://microvesicles.org/) [152,153]. EVpedia is the only one of the three databases which includes eukaryotic and prokaryotic extracellular vesicles data (http://student4.postech.ac.kr/evpedia2_xe/xe/) [154,155]. In the work described here, only human proteomes were considered.

The exosomal protein cargo for each biofluid was refined through several steps (Figure 1), considering the following: number of entries for the specific biofluid, number of entries for *H. sapiens*, number of entries for *H. sapiens* and exosomes, number of ID entries for gene names of *H. sapiens* and exosomes. Finally, repeated gene names were removed to obtain the final biofluid gene list. The EVpedia output was in the form of Uniprot IDs and thus it was necessary to convert this data to gene names. To achieve this the Uniprot Retrieve/ID mapping tool was used to convert the Uniprot IDs to gene names (https://www.uniprot.org/uploadlists/; accessed on 14 January 2020).

The proteome list for each biofluid includes every protein/corresponding gene name present in at least one of the databases. In the case of CSF, only data from EVpedia was collected since no information was available in the other databases.

Additionally, the proteomes obtained from the databases were complemented by including information from a literature search on PubMed (https://PubMed.ncbi.nlm.nih.gov/; accessed on 17 January 2020) using the keywords: “exosomes and Alzheimer´s disease and CSF or Serum or Plasma” (Supplementary Table S4). The protein name was converted to gene name, as described above. Thus, the final proteome lists for each biofluid included information collected from the databases and from the literature search (Supplementary Tables S5–S7). These final lists will be herein referred to as the distinct biofluid proteomes.

### 3.2. Proteome GO and Overlap Analysis 

All proteomes were analyzed using the Panther classification system (http://www.pantherdb.org/), version 14. Gene ontology (GO) enrichment analysis at the molecular function, biological process was carried out on the 22 January 2020. The biofluids proteomes’ main pathways were likewise identified.

The proteomes of the different biofluids, were compared and overlapped against each other by means of a Venn diagram using the Bioinformatics and Evolutionary Genomics website (http://bioinformatics.psb.ugent.be/webtools/Venn/; accessed on 30 January 2020). This allowed identifying the shared biofluids proteome, that is also present in CSF.

### 3.3. Aβ-binding Proteins Literature Search

To identify the Aβ-binding proteins present in the group of 258 exosomal proteins shared by the three biofluids (CSF, serum and plasma), an individual PubMed search was performed for each protein using the following keywords “(protein name) and Abeta and (binding or clearance or aggregating or fibril formation)” or “(gene name) and Abeta and (binding or clearance or aggregating or fibril formation)”.

### 3.4. EVs Isolation and Characterization

Human samples were obtained from participants of the pcb-cohort, a primary care-based cohort stablished by the group, which includes controls and individuals with dementia, characterized by cognitive testing such as clinical dementia rating (CDR) and Mini-Mental State Examination (MMSE) [156,157]. CDR scale applied scores between 0 and 3 where 0 accounts for normal, 1 for mild dementia, 2 for moderate and 3 for severe dementia stages. The MMSE scale considered scores between 0 and 30 and the cutoffs were set according to the Portuguese population: 0–2 years of literacy, cutoff = 22; 3–6 years of literacy, cutoff = 24; and ≥7 years of literacy, cutoff = 27. Tests equal to or below cutoff were scored to cognitive deficits and those above were scored as normal. In this study, controls (*n* = 32; CDR = 0 and that scored negative for MMSE (MMSE–); mean age 76.69 ± 8.07) and demented individuals (*n* = 32; CDR ≥ 1 and that scored positive for MMSE (MMSE+); mean age 77.38 ± 9.17) were included. AD clinical diagnosed cases were also monitored, 8 AD patients that were CDR ≥ 1 and MMSE+, plus 1 AD patient who scored CDR = 1 (mean age 78.67 ± 5.07); and 9 age- and sex-matched controls (mean age 77.56 ± 4.83), Supplementary Table S2. The study is part of a project approved by the Ethics Committee (Comissão de Ética para a Saúde da ARS Centro, protocol No. 012804-04.04.2012) and by the National Committee for Data Protection (CNPD No. 369/2012). Serum from controls (*n* = 12; mean age 67.58 ± 7.74) and AD cases (*n* = 12; mean age 73.17 ± 10.66) were also obtained from the UMG-cohort, established at the University Medical Center of Goettingen, Germany. These individuals were highly characterized including either cognitive testing, CSF-neurochemical diagnostic biomarkers, PET imaging, or in combination, as described in [9], Supplementary Table S3. The collection of these samples and their use was approved by the ethics committee of the University of Goettingen (9/2/16). All participants gave written informed consent.

Serum-derived EVs, with exosome-like characteristics, were isolated from 250 µL of serum from controls and individuals with either dementia, AD, or both, cases using the ExoQuick Serum Exosome Precipitation Solution (System Biosciences, Palo Alto, CA, USA). The resulting exosomal pellet was resuspended in RIPA buffer (Sigma-Aldrich) with protease inhibitors for Western blot and ELISA; or in PBS for NTA analysis, as previously described [9,147].

Randomly chosen samples were used for NTA analysis and Western blot procedures. NTA analyses were performed using a Nanosight NS300 (Malvern Instruments, Malvern, UK) and NTA 3.2 software (Malvern Instruments, Malvern, UK). NTA analysis was then carried out in duplicate for each sample. Particle numbers obtained were multiplied by the dilution factor to determine particle concentration.

A set of exosomal markers was also tested for exosome characterization. After bicinchoninic acid assay (BCA) protein quantification assay, normalized samples for protein content (50 µg of protein) were used for SDS-polyacrylamide gel electrophoresis (SDS-PAGE), followed by a wet electrophoretic transfer of the proteins. Nitrocellulose membranes were blocked in non-fat dry milk solution (5%) and incubated overnight with the exosomal marker antibodies, anti-TSG101 (1:500) (612,697; BD transduction laboratories, San Jose, CA, USA) and anti-HSP70 (1:200) (sc-24; Santa Cruz Biotechnology, Dallas, TX, USA) or the negative exosomal marker antibody anti-calnexin (1:500) (ADI-SPA-860-J; Enzo Life Sciences, Farmingdale, NY, USA). This was followed by incubation with the anti-mouse IgG, HRP-linked antibody (1:2000) (Cell Signaling Technology, Danvers, MA, USA) or anti-rabbit IgG, HRP-linked antibody (1:2000) (7074; Cell Signaling Technology, Danvers, MA, USA). Protein bands were detected with the Chemidoc gel imaging system (Bio-Rad, Hercules, CA, USA) with Image Lab Touch Software (Bio-Rad, Hercules, CA, USA).

### 3.5. Evaluation of Gelsolin Levels 

For GSN level evaluation, an equal amount of total protein (50 µg) was likewise loaded from each sample. An exosomal pool was included in each membrane for sample normalization between membranes. SDS-PAGE was carried out and the proteins were electrophoretic transferred to nitrocellulose membranes in a wet system, as described above. The membranes were blocked and incubated overnight with the primary monoclonal antibody, anti-gelsolin (1:500) (G4896; Sigma-Aldrich). Then, the membranes were incubated with the secondary anti-mouse IgG, HRP-linked antibody (1:5000) (Cell Signaling Technology, Danvers, MA, USA). To detect the protein bands, chemiluminescence reagent ECL Select (GE Healthcare Life Sciences, Chicago, IL, USA) was added to the membranes and images were obtained using Chemidoc gel imaging system (Bio-Rad, Hercules, CA, USA) with Image Lab Touch Software (Bio-Rad, Hercules, CA, USA). Further, gelsolin levels were also accessed in serum-derived exosomes using the commercial Human Gelsolin ELISA Kit (ab270215; Abcam, Cambridge, UK) following the manufacturer’s instructions. Samples were diluted (1:100) and equal amounts of total protein (0.5 µg) for each sample were added for GSN quantification by ELISA.

### 3.6. Statistical Analysis 

The Shapiro–Wilk test was used to assess the data distribution. Particle concentrations, mode sizes, cognitive scores, CSF biomarker results and median gelsolin levels between controls and the dementia group (CDR ≥ 1 and MMSE+) or controls and AD were compared, using the Mann–Whitney test, since data did not follow a normal distribution. In the case of gelsolin, an exosomal pool sample was used to normalize data across results either in Western blot or ELISA assays. For immunoblotting, the densitometry value of each individual band was divided by the densitometry value of the pool within the same membrane. These ratios were expressed as arbitrary units (A.U.). In ELISA assays, the mean concentration obtained for each individual was divided by the mean concentration of the exosomal pool within the same ELISA plate and then multiplied by the mean concentrations of all pools, from all plates. The level of significance was set at or below 5%. Analyses were performed using GraphPad Prism 7 (GraphPad Software, La Jolla, CA, USA) or SPSS version 22 (IBM).

## 4. Conclusions

Exosome content can reflect physiological and pathological stages highlighting the potential of these EVs as biomarkers resources for disease diagnostics or therapeutics. This is particularly relevant in diseases such as AD, where the only molecular available methodology is based on monitoring of a biomarker triplet in the CSF, which requires a lumbar puncture, limiting its use as a widely available tool, hence identification of peripheral biomarker is of clinical value.

Biomarkers that enter the bloodstream, in particular those arising from the brain will be considerably diluted, but exosomes may represent a contained source of protein candidates. Exosomes are innovative tools with huge biomarker potential in AD, not only because these nanovesicles may represent an enclosed source of biomarker candidates, but also because exosomes arising from peripheral biofluids can be more accessible and widely available. Besides GSN, the bioinformatic analysis carried out revealed several exosomal putative biomarker candidates, some already linked to AD that can be further tested for their differential and diagnostic potential.

## Figures and Tables

**Table 1:** Aβ-binding proteins found in the shared exosomal proteome and their expression tendency in AD. Gene and protein names of Aβ-binding proteins identified in the exosomes isolated from CSF, serum and plasma are indicated in the table, as well as the respective alterations reported in AD.

Gene	Protein Name	CSF	Serum	Plasma	Brain	Publication
A2M	Alpha-2-macroglobulin		↑/=	↑/=		[24,25,26]
ALB	Albumin	=	=			[26,27,28,29]
APCS	Serum amyloid P-component	=	=		↑	[30,31,32]
APOA1	Apolipoprotein A-I	=	↓	↓/=	=	[33,34,35,36,37,38,39,40,41,42,43]
APOA2	Apolipoprotein A-II		↓	↓/=		[34,35,38,42]
APOA4	Apolipoprotein A-IV			↑		[41]
APOB	Apolipoprotein B-100			↓/=/↑		[35,43,44]
APOC1	Apolipoprotein C-I			=	↓	[42,45]
APOC3	Apolipoprotein C-III			↓		[43]
APOE	Apolipoprotein E	=/↑		↑/=	↓/=	[33,39,40,43,46,47]
BCHE	Cholinesterase	=	↓		↑	[48,49,50]
C1QA, C1QB and C1QC	Complement C1q	↓*		↑**		[51,52,53]
C3	Complement C3	↑			↑	[54,55]
C4BPα	C4b-binding protein alpha chain	=*		=*/↓		[35,56]
CAT	Catalase			=	↑/=	[57,58,59]
CFH	Complement factor H		↓	=	=	[60,61,62,63]
CFI	Complement factor I				=	[63]
CLU	Clusterin	↑		↑/=	↑/=	[33,35,39,46,47,64,65,66,67]
CSTB	Cystatin-B					Not found
F12	Coagulation factor XII	↑		=		[68]
FGB	Fibrinogen beta chain			↑		[44]
GAPDH	Glyceraldehyde-3-phosphate dehydrogenase			↑**	↑	[69,70]
GC	Vitamin D-binding protein			↓		[35,44]
GSN	Gelsolin	↓		↓/↑	=/↓	[35,71,72,73,74]
HP	Haptoglobin	↑	↑/=			[26,75,76,77,78]
HSPB1	Heat shock protein beta-1				↑	[79]
IGHM	Immunoglobulin heavy constant mu			↑		[35]
L1CAM	Neural cell adhesion molecule L1			=***		[19,80]
LRP1	Prolow-density lipoprotein receptor-related protein 1			↓	↑/↓	[81,82,83,84]
NCL	Nucleolin				↓	[85]
PFN1	Profilin-1					Not found
PZP	Pregnancy zone protein		↑		↑	[86,87]
RELN	Reelin	↑		=	↑	[88,89,90]
S100A8	Protein S100-A8		↓			[91]
S100A9	Protein S100-A9	↓	↓	=		[91,92,93]
SELENOP	Selenoprotein P	↑			↑	[94]
SERPINA1	Alpha-1-antitrypsin	↑/=	↑/=	↑/=		[26,42,67,95,96]
SERPINA3	Alpha-1-antichymotrypsin, ACT	↑/=	↑/=	↑	↑	[95,97,98,99,100,101,102,103,104,105,106,107,108,109,110,111,112,113,114]
TF	Serotransferrin	=		=		[115,116]
THBS1	Thrombospondin-1		↓		↓	[91,117]
TTR	Transthyretin	↓/=	↓	↓/↑		[35,118,119,120,121,122]
TUBB	Tubulin				↓*	[70]

* For C1QA, C1QB and C1QC, articles where C1Q levels were assessed were considered. The same happened for the tubulin β chain and C4BPα chain, where tubulin or C4BP levels were considered, independently of the chain. ** Levels of S-glutathionylated GAPDH form were reported. *** Plasma-derived exosomes. ↑, increased; ↓, decreased; =, no change.

## Data Availability

Not applicable.

## Supplementary Materials

The following are available online at https://www.mdpi.com/article/10.3390/ijms22083933/s1, Figure S1: Serum-derived EVs characterization; Figure S2: Western blot analysis of gelsolin in UA- and UMG-groups; Table S1: Aβ-binding proteins found in exosomal proteomes from CSF, serum and plasma; Table S2: Demographics and clinical data of UA-group participants; Table S3: Demographics and clinical data of UMG-group participants, Table S4: List of proteins found in exosomes isolated from CSF, serum and plasma and previously related to Alzheimer’s disease; Table S5: Final CSF proteome list; Table S6: Final serum proteome list; Table S7: Final plasma proteome list.
