# Supplementary material for: Exosomal Aβ-Binding Proteins Identified by “In Silico” Analysis Represent Putative Blood-Derived Biomarker Candidates for Alzheimer´s Disease
Source: Int J Mol Sci. 2021 Apr 11;22(8):3933. doi: 10.3390/ijms22083933 (PMC8070602; doi:10.3390/ijms22083933)
Supplement: Supplementary file 1 [file ijms-22-03933-s001.pdf]

International Journal of *Molecular Sciences*

MDPI

Supplementary Figure S1

A. B. C. D.

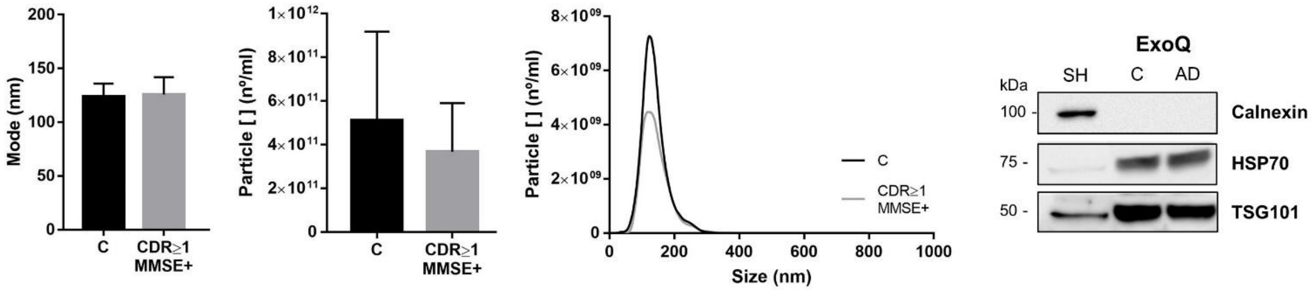

Supplementary Figure S1. Serum-derived EVs characterization. Particle concentration (**A**), mode sizes (**B**) and size distribution curves (**C**) were assessed by Nanoparticle Tracking Analysis. Negative exosomal marker Calnexin and exosomal markers HSP70 and TSG101 were assessed through western blot analysis (**D**). Error bars indicates standard deviations. Abbreviations: AD, Alzheimer's Disease; C, Controls; CDR, Clinical Dementia Rating; MMSE, Mini-Mental State Examination; SH, SH-SY5Y cell lysates.

Supplementary Figure S2

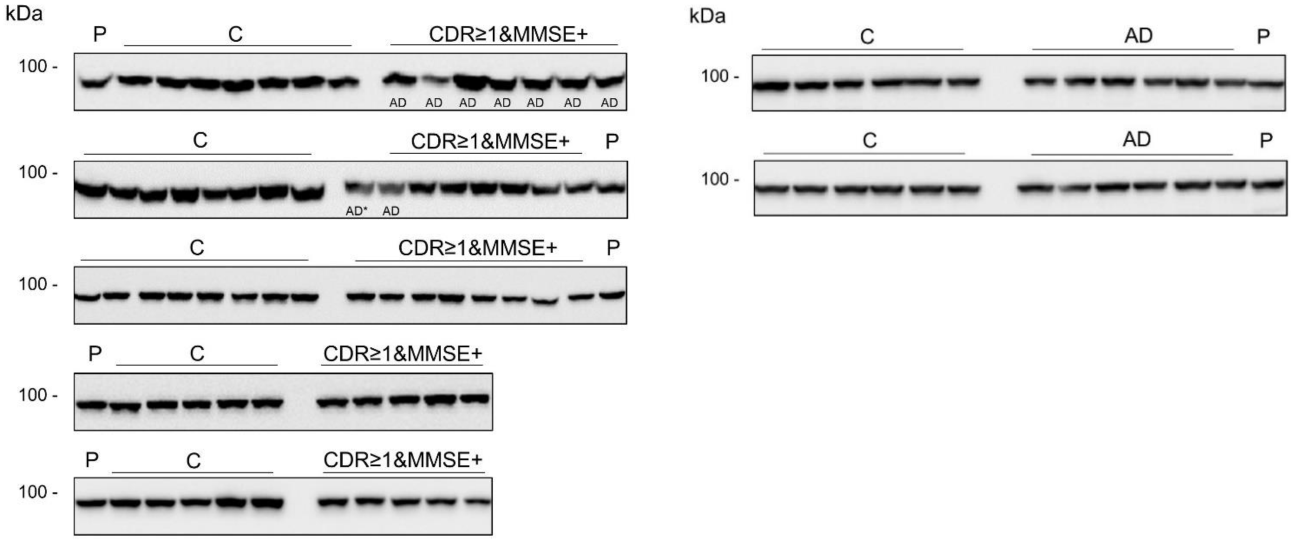

(**a**) (**b**)

Supplementary Figure S2. Western blot analysis of gelsolin in UA- and UMG-groups. Gelsolin levels were assessed in serum-derived exosomes of Controls and individuals with dementia from UA-dementia group (CDR≥1&MMSE+; *n* =3 2) (**A**), which includes AD clinically diagnosed cases (indicated as AD). One clinical diagnosed AD case that scored CDR = 1 but was negative for MMSE (AD*), was also included in analyses for comparison. Gelsolin levels were also assessed in serum-derived exosomes of Controls and AD cases (*n* = 12) from UMG-group (**B**). Exosomal pool (P) was used for data normalization. In all blots gelsolin bands appeared at ~ 90 kDa, as expected. Abbreviations: AD,

Alzheimer's disease; C, Controls; CDR, Clinical Dementia Rating; MMSE, Mini-Mental State Examination; P, Exosomal pool.

Supplementary Table S1. Aβ-binding proteins found in exosomal proteomes from CSF, serum and plasma. References reporting the Aβ binding to each protein are indicated.

Gene name	Protein names	References
A2M	Alpha-2-macroglobulin	[1,2]
ALB	Albumin	[3–5]
APCS	Serum amyloid P-component	[6–8]
APOA1	Apolipoprotein A-I	[9–11]
APOA2	Apolipoprotein A-II	[11]
APOA4	Apolipoprotein A-IV	[12]
APOB	Apolipoprotein B-100	[10]
APOC1	Apolipoprotein C-I	[13]
APOC3	Apolipoprotein C-III	[10]
APOE	Apolipoprotein E	[10,11,14,15]
BCHE	Cholinesterase	[16,17]
C1QA, CQ1B and C1QC	Complement C1q	[18,19]
C3	Complement C3	[20–22]
C4BPA	C4b-binding protein alpha chain	[23]
CAT	Catalase	[24–26]
CFH	Complement factor H	[27]
CFI	Complement factor I	[28]
CLU	Clusterin	[11,29–32]
CSTB	Cystatin-B	[33]
F12	Coagulation factor XII	[34]
FGB	Fibrinogen beta chain	[35]
GAPDH	Glyceraldehyde-3-phosphate dehydrogenase	[36]
GC	Vitamin D-binding protein	[37]
GSN	Gelsolin	[38,39–41]
HP	Haptoglobin	[42]
HSPB1	Heat shock protein beta-1	[43,44]
IGHM	Immunoglobulin heavy constant mu	[45]
L1CAM	Neural cell adhesion molecule L1	[46]
LRP1	Prolow-density lipoprotein receptor-related protein 1	[47–50]
NCL	Nucleolin	[51]
PFN1	Profilin-1	[52]
PZP	Pregnancy zone protein	[53]
RELN	Reelin	[54,55]

S100A8	Protein S100-A8	[56]
S100A9	Protein S100-A9	[57]
SELENOP	Selenoprotein P	[58,59]
SERPINA1	Alpha-1-antitrypsin	[60]
SERPINA3	Alpha-1-antichymotrypsin	[60–62]
TF	Serotransferrin	[63]
THBS1	Thrombospondin-1	[64]
TTR	Transthyretin	[65,66]
TUBB	Tubulin beta chain	[67,68]

Supplementary Table S2. Demographics and clinical data of UA-group participants.

	**UA-group**					
	**C (N=32)**	**CDR≥1 & MMSE+ (N=32)**	**P-value**	**C (N=9)**	**AD (N=9)**	**P-value**
**Age** (mean±SD)	76.69±8.07	77.38±9.17	0.58	77.56±4.83	78.67±5.07	0.99
**Years of literacy** (mean±SD)	5.96±4.73	3.38±3.24	0.07	6.67±5.94	3.33±2.69	0.05
**MMSE scores** (mean±SD)	28.38±1.79	17.41±3.88	<0.01	28.89±1.69	15.00±3.61	0.11

Abbreviations: AD, Alzheimer's disease cases; C, Controls; CDR, Clinical Dementia Rating; MMSE, Mini-Mental State Examination; P, p-value, SD, Standard deviation.

Supplementary Table S3. Demographics and clinical data of UMG-group participants.

	**UMG-group**		
	**C (N=12)**	**AD (N=12)**	**P-value**
**Age** (mean±SD)	67.58±7.74	73.17±10.66	0.1163
**Years of literacy** (mean±SD)	12.75±3.99	11.50±1.64	0.82*
**MMSE scores** (mean±SD)	26.42±2.84	24.83±3.24	0.18
**CDT** (points) (mean±SD)	2.58±1.08	3.08±1.38	0.26
**CSF Aβ1-42** (ng/ml) (mean±SD)	1029±461.7	592.0±204.1	<0.01
**CSF Aβ1-40** (ng/ml) (mean±SD)	12670±6362	17274±5542	0.08
**CSF Aβ1-42/1-40** (mean±SD)	0.07±0.007	0.03±0.006	<0.001
**CSF P-Tau 181** (pg/ml) (mean±SD)	277.7±129.1	695.0±336.9	<0.001
**CSF Tau** (pg/ml) (mean±SD)	43.33±15.65	86.98±31.79	<0.001

Abbreviations: AD, Alzheimer's disease cases; C, Controls; CDT, Clock-Drawing Test; CSF, Cerebrospinal fluid; MMSE, Mini-Mental State Examination; P, p-value, SD, Standard deviation. * Data available only for n=8 Controls and n=6 ADs.

Supplementary Table S4. List of proteins found in exosomes isolated from CSF, Serum and Plasma and previously related to Alzheimer's disease. These proteins were not found in the EVpedia, EXOCARTA and Vesiclepedia proteomes but due to the literature reported association with AD, these were added to the exosomal proteome lists obtained from the databases.

	**Proteins identified in the literature**	
	**Gene names**	**Reference**
**CSF**	MAPT	[69]
**Serum**	L1CAM	[70]
	SNAP-25	[70]
	TSG101	[70]
**Plasma**	APH1A	[71]
	APP	[71–74]
	BACE1	[71]
	CD46	[75]
	CD55	[75]
	CD59	[75]
	CD93	[75]
	CFB BF	[75]
	CR1	[75]
	CSPG4	[76]
	CTSD	[77]
	ENO2	[71,77]
	FGF13	[76]
	FGF2	[76]
	GAP43	[74]
	GDNF	[71]
	Gfap	[71,75]
	GLUL	[71,75,76]
	GRIA4	[76,78]
	HGF	[76]
	HSP72*	[77]
	IGF1	[76]
	IL1B	[75]
	IL6	[75]
	Irs1	[73]
	L1CAM	[72–74,77,78]
	Lamp1	[77]
	MAPT	[71–74]
	MOG	[76]
	NCAM1	[73,77]
	NCSTN	[71]
	NEFL	[71,73,76,77]
	NLGN1	[78]

	Nptx2	[76,78]
	Nrgn	[72,74]
	NRXN2	[74]
	PDCD6IP	[73]
	PDGFRA	[76]
	PSEN1	[71]
	PSENEN	[71]
	REST	[72]
	SEPTIN8	[71]
	SLC1A3	[71,75]
	SYN1	[74]
	Synpo	[74]
	Syp	[73,74]
	SYT2	[74]
	TSG101	[73]

Supplementary Table S5. Final CSF Proteome List. List of CSF exosomal gene names obtained from overlap of the EVpedia, EXOCARTA and Vesiclepedia. Exosome gene names mined from the literature were also included.

Proteome of CSF-derived exosomes - Gene name							
A1BG	CACNA2D2	DNAJC5	HDHD2	KRT6C	NUDC	RALA	SLC5A5
A2M	CAD	DNM1	HEBP1	KRT7	NUP133	RAN	SLC9A3R1
AARS	CADM1	DPP6	HERC1	KRT73	NUP62	RAP1A	SLIT1
AATF	CADM2	DPP7	HEXA	KRT77	NUTF2	RAP2A	SLITRK1
ABHD12B	CADM3	DPYSL2	HIBADH	KRT78	OAF	RARRES2	SLITRK2
ABI3BP	CALM1	DRG2	HINT1	KRT79	OGN	RBBP4	SLITRK4
ABR	CALML5	DSC1	HIST2H2BF	KRT8	OLFM1	RBBP7	SLITRK5
ACADM	CALR	DSG1	HK1	KRT80	OLFML2A	RBM8A	SLPI
ACADS	CAMK2A	DSP	HLA-A	KRT81	OLFML3	RBMX	SMC3
ACAT2	CANT1	DYNC1H1	HLA-B	KRT83	OMD	RBP3	SMOC1
ACLY	CAP1	DYNC2H1	HLA-DRA	KRT85	OMG	RBP4	SNAP25
ACO2	CAPG	ECM1	HLA-DRB1	KRT86	OPA1	RCC1	SNED1
ACOT7	CAPZA1	ECM2	HMCN1	KRT9	ORM1	RCC2	SNRNP40
ACP1	CAPZB	EDIL3	HMGB1	L1CAM	ORM2	RELN	SOD1
ACP2	CASP14	EEF1A1	HMGB2	LAMA2	OXCT1	RFC2	SOD2
ACTC1	CAT	EEF1D	HNRNPA0	LAMA4	P4HB	RFC3	SOD3
ACTG1	CBLN1	EEF1G	HNRNPA1	LAMA5	PA2G4	RFC4	SORCS1
ACTN4	CBLN3	EEF2	HNRNPA2B1	LAMB1	PAK2	RGMA	SPARC
ACTR2	CBR1	EFEMP1	HNRNPA3	LAMB2	PAM	RGMB	SPARCL1
ADA	CBX1	EFEMP2	HNRNPAB	LAMC1	PAPLN	RHOA	SPOCK3
ADAM15	CCT2	EFHD2	HNRNPC	LAMC3	PARK7	RIDA	SPON1
ADAM22	CCT3	EFTUD2	HNRNPD	LBP	PARP1	RIPOR3	SPP1
ADAR	CCT4	EIF1	HNRNPDL	LCAT	PCBP1	RNASE1	SPRR1B

ADCY3	CCT5	EIF1AY	HNRNPF	LCN1	PCBP2	RNASE4	SPRR2G
ADGRB1	CCT6A	EIF3D	HNRNPH1	LCP1	PCDH17	RNF40	SPTA1
ADGRB2	CCT7	EIF3G	HNRNPH3	LDHA	PCDH9	RNH1	SPTAN1
ADGRL1	CCT8	EIF3H	HNRNPK	LDHB	PCDHAC2	ROBO1	SPTBN1
ADH5	CD109	EIF3M	HNRNPL	LEFTY2	PCDHGC3	RPA1	SPTBN2
ADSL	CD14	EIF4A1	HNRNPM	LFNG	PCMT1	RPL10A	SRI
AEBP1	CD2AP	EIF4A2	HNRNPU	LGALS1	PCNA	RPL12	SRM
AFM	CD44	EIF4A3	HNRNPUL1	LGALS3BP	PCOLCE	RPL13	SRP14
AGRN	CD47	EIF4G1	HP	LGALS7	PCSK1N	RPL13A	SRPX
AGT	CD59	EIF5A	HPR	LINGO1	PCSK2	RPL14	SRRT
AHCY	CD5L	ELMO1	HPRT1	LIPN	PCSK9	RPL17	SRSF5
AHNAK	CD81	EMILIN1	HPX	LMAN1	PCYOX1	RPL18	SSB
AHSG	CD9	EMILIN2	HRG	LMAN2	PDCD6	RPL18A	ST13
AK1	CDC42	ENO1	HRNR	LMNB1	PDCD6IP	RPL21	ST8SIA3
AKR1B1	CDC73	ENO2	HS6ST3	LOR	PDIA3	RPL23	ST8SIA5
ALB	CDH1	ENPP2	HSD17B10	LOXHD1	PDLIM1	RPL23A	STAB1
ALDH9A1	CDH13	ENPP6	HSP90AA1	LOXL1	PDYN	RPL24	STIP1
ALDOA	CDH15	EPHA4	HSP90AB1	LPA	PEBP1	RPL29	STMN1
ALDOC	CDH2	EPHB6	HSP90B1	LRG1	PEPD	RPL3	STOML2
ALOX12B	CDH5	EPRS	HSPA13	LRP1	PF4V1	RPL36A	STRAP
AMBP	CDK3	ERAP1	HSPA1A	LRRC4B	PFDN2	RPL37A	STX1A
AMY1A	CDK5RAP2	ERAP2	HSPA2	LRRFIP1	PFKP	RPL38	STX1B
ANG	CDSN	ESD	HSPA4	LSM12	PFN1	RPL4	STXBP1
ANGPTL2	CELSR2	ETF1	HSPA5	LSM14A	PFN2	RPL5	SUMO3
ANP32B	CEMIP	ETFA	HSPA8	LSM8	PGAM1	RPL6	SUPT16H
ANXA1	CETP	EXOSC7	HSPA9	LTA4H	PGK1	RPL7	SYN1
ANXA11	CFD	EXTL2	HSPB1	LTBP1	PGLS	RPL7A	SYNGR1
ANXA2	CFH	EZR	HSPD1	LTBP2	PGLYRP2	RPL8	SYT1
ANXA4	CFHR1	F10	HSPE1	LTBP4	PGM1	RPL9	TAGLN
ANXA5	CFHR2	F12	HSPG2	LTF	PGRMC1	RPLP0	TAGLN2
ANXA6	CFI	F13A1	HSPH1	LUC7L	PHB	RPLP2	TALDO1
ANXA7	CFL1	F13B	HTRA1	LUC7L2	PHB2	RPN1	TARSL2
AP2A1	CHAD	F2	HVCN1	LUM	PHGDH	RPS10P5	TCEA1
AP2B1	CHGA	F5	IARS	LY6H	PI4KA	RPS11	TCN2
APCS	CHGB	F9	ICAM5	LYN	PIGR	RPS13	TCP1
APEH	CHI3L1	FABP5	IDH2	LYNX1	PIP	RPS15	TF
APIP	CHL1	FAM20A	IDH3B	LYZ	PITRM1	RPS15A	TFRC
APLP1	CHMP2A	FAM3C	IDS	M6PR	PKM	RPS17	TGFBI
APLP2	CHRD	FARSA	IFI16	MAMDC2	PKP1	RPS18	TGFBR3
APMAP	CHRDL1	FASN	IFT140	MAN1A1	PLD3	RPS19	TGM1
APOA1	CHST10	FAT2	IFT172	MAN1B1	PLD4	RPS2	TGM3
APOA2	CHST15	FBL	IFT74	MAN1C1	PLG	RPS20	THADA

APOA4	CISD1	FBLN1	IFT80	MAN2A1	PLOD1	RPS21	THBS1
APOB	CKB	FBLN2	IFT81	MAN2A2	PLOD3	RPS23	THBS2
APOC1	CKM	FBLN5	IFT88	MAN2B1	PLP1	RPS25	THBS4
APOC2	CKMT1A	FBLN7	IGF2	MANBA	PLTP	RPS26	THSD4
APOC3	CLEC11A	FBN1	IGF2R	MAP3K2	PLXDC2	RPS27	THY1
APOD	CLEC3B	FCER1G	IGFALS	MAPK1	PLXNB2	RPS28	TIMP1
APOE	CLIC6	FCGBP	IGFBP2	MAPT	PNP	RPS3	TIMP2
APOF	CLSTN1	FCGR1A	IGFBP4	MARCKS	POMGNT1	RPS3A	TIMP3
APOH	CLSTN2	FCN2	IGFBP6	MASP1	PON1	RPS4X	TKT
APOL1	CLSTN3	FCN3	IGFBP7	MAT2A	PON3	RPS5	TLN1
APOM	CLTC	FETUB	IGHA1	MATN2	POTEE	RPS8	TMEM132A
APP	CLU	FGA	IGHA2	MATN3	PPBP	RPSA	TMEM132D
ARF3	CLUAP1	FGB	IGHD	MB	PPIA	RRAS	TMEM198
ARF4	CNDP1	FGFR1	IGHG1	MBP	PPIB	RSL1D1	TMPO
ARF5	CNDP2	FGG	IGHG2	MCAM	PPIC	RUVBL2	TMSB4X
ARF6	CNN3	FHL1	IGHG3	MCM2	PPP1R11	RYR2	TNR
ARG1	CNP	FILIP1L	IGHG4	MCM3	PPP2CB	S100A16	TNXB
ARHGDIA	CNTN1	FKBP1A	IGHM	MCM4	PPP2R1A	S100A6	TOMM22
ARHGDIB	CNTN2	FKBP3	IGHV1-2	MCM6	PPP2R2A	S100A7	TPI1
ARL3	CNTN4	FKBP4	IGHV1-46	MDC1	PPP5C	S100A8	TPM2
ARMC9	CNTN5	FLG	IGHV3-13	MDH1	PRCP	S100A9	TPM3
ARPC1B	CNTN6	FLG2	IGHV3-23	MDH2	PRDX1	SAA4	TPP1
ARPC2	CNTNAP2	FLNA	IGHV3-33	MEGF10	PRDX2	SAE1	TPT1
ARPC4-TTLL3	CNTNAP4	FLNB	IGHV3-48	MEGF8	PRDX3	SARNP	TRA2B
ART3	COL12A1	FMOD	IGHV3-7	MET	PRDX5	SART1	TRHDE
ART4	COL14A1	FN1	IGHV4-59	METRNL	PRDX6	SBSN	TRIM33
ASAH1	COL15A1	FOLR1	IGKC	MFGE8	PRELP	SCG2	TTC21B
ATIC	COL16A1	FOLR2	IGKV1-17	MGAT1	PREP	SCG3	TTC30B
ATP1A1	COL18A1	FRZB	IGKV1-39	MGAT2	PRG4	SCG5	TTN
ATP1A2	COL1A1	FSCN1	IGKV1-5	MGAT5	PRNP	SCRG1	TTR
ATP1A3	COL1A2	FSTL1	IGKV1D-12	MGP	PROC	SCUBE2	TUBA1A
ATP1B1	COL3A1	FSTL4	IGKV1D-33	MIA	PROS1	SDCBP	TUBA1B
ATP1B2	COL4A1	FSTL5	IGKV2-30	MIF	PROZ	SDF2L1	TUBA1C
ATP2A2	COL4A2	FTH1	IGKV2D-28	MINPP1	PRPF19	SDF4	TUBA4A
ATP2B1	COL6A1	FUCA1	IGKV3-15	MMP2	PRPF4	SELENBP1	TUBB
ATP2B2	COL6A2	FUCA2	IGKV3-20	MOV10	PRPF40A	SELENOP	TUBB2A
ATP2B3	COL6A3	G6PD	IGLC2	MPO	PRPS2	SELPLG	TUBB3
ATP4A	CORO1A	GAA	IGLL5	MPST	PRR4	SEMA3B	TUBB4A
ATP5F1A	CORO1C	GALK1	IGLV1-40	MSLN	PRRT2	SEMA3C	TUBB4B
ATP5F1B	COTL1	GALNT10	IGLV1-47	MSN	PRSS1	SEMA3G	TUFM
ATP5F1D	COX4I1	GALNT13	IGLV1-51	MTA3	PRSS3	SEMA4B	TWF2
ATP5PO	CP	GALNT15	IGLV2-14	MT-CO2	PSAP	SEMA6A	TXN

ATP6AP1	CPA4	GALNT2	IGLV3-19	MTHFD1	PSMA1	SEMA6D	TXNDC17
ATP6AP2	CPB2	GALNT5	IGLV3-21	MUC5B	PSMA2	SEMG1	TXNRD1
ATP6V0A1	CPE	GALNT6	IGLV3-25	MYH2	PSMA3	SEMG2	UBA1
ATP6V0D1	CPN1	GALNT7	IGLV7-43	MYH4	PSMA5	SEPTIN7	UBA2
ATP6V1A	CPN2	GANAB	IGSF8	MYH7B	PSMA6	SERBP1	UBC
atpE	CPQ	GAPDH	IL6ST	MYH9	PSMA7	SERPINA1	UBE2O
ATRN	CPVL	GAS6	ILF2	MYL12B	PSMB1	SERPINA10	UBQLN4
AZGP1	CPXM2	GASK1B	IMMT	MYL6	PSMB10	SERPINA12	UCHL5
B2M	CPZ	GBA	IMPA1	MYO1G	PSMB3	SERPINA3	UGGT1
B3GALNT1	CRABP1	GC	IMPAD1	MYO5A	PSMB4	SERPINA4	UQCRC1
B3GAT3	CROCC	GDA	IMPDH2	MYOC	PSMB5	SERPINA5	USP14
B3GNT2	CRP	GDF11	INA	NAA15	PSMB6	SERPINA6	USP9Y
B3GNT9	CRTAC1	GDI1	IPO5	NAPA	PSMB9	SERPINA7	VAMP2
B4GALNT1	CRYBG1	GFPT1	IQGAP1	NASP	PSMC2	SERPINB1	VAMP5
B4GALT1	CRYM	GGCT	ISLR	NCAM1	PSMC3	SERPINB12	VASN
B4GAT1	CSDE1	GGH	ITGAM	NCAM2	PSMD11	SERPINB3	VAT1
BANF1	CSPG4	GLRX	ITGB1	NCAN	PSMD13	SERPINB5	VCAM1
BCAN	CST3	GLUD1	ITGB2	NCAPH	PSMD2	SERPINB6	VCAN
BCHE	CST4	GM2A	ITIH1	NCCRP1	PSMD7	SERPINC1	VCL
BCL2L2	CST6	GMPS	ITIH2	NCKAP1	PTBP1	SERPIND1	VCP
BGN	CSTA	GNAI1	ITIH3	NCL	PTGDS	SERPINE2	VDAC1
BLMH	CSTB	GNAI2	ITIH4	NDRG2	PTK7	SERPINF1	VDAC2
BPIFB1	CTSA	GNAO1	ITIH5	NDRG4	PTMA	SERPINF2	VDAC3
BSG	CTSB	GNB1	ITPR1	NDUFS8	PTN	SERPING1	VGF
BTD	CTSD	GNB2	JAM3	NEFL	PTPRC	SERPINI1	VIM
BTF3	CTSF	GNPTG	JCHAIN	NEFM	PTPRD	SET	VPS26B
BUB3	CTSH	GNS	JPT1	NEGR1	PTPRF	SEZ6	VSNL1
C16orf89	CTSL	GOT1	JUP	NELL2	PTPRN	SEZ6L	VTN
C1QA	CUTA	GOT2	KATNA1	NEO1	PTPRN2	SEZ6L2	VWF
C1QB	CYB5B	GPI	KCNJ13	NEU1	PTPRS	SF3A1	WARS
C1QC	CYBB	GPLD1	KCTD12	NFASC	PTPRZ1	SF3A3	WBP2
C1QTNF3	CYSTM1	GPM6A	KEL	NID1	PURB	SF3B1	WDR1
C1QTNF5	DAG1	GPX3	KIAA0319L	NID2	PXDN	SF3B3	WDR35
C1R	DARS	GRIA4	KIF21A	NLGN2	PYGB	SF3B4	WFIKKN2
C1RL	DAZAP1	GSN	KIF21B	NME2	PYGM	SFN	XP32
C1S	DCC	GSR	KIF3C	NNT	PZP	SFPQ	XPNPEP1
C2	DCD	GSTM3	KLK6	NOP58	QDPR	SGCE	XPO1
C3	DCN	GSTO1	KLKB1	NOTCH3	QPCT	SGSM2	XPOT
C4B	DCTN1	GSTP1	KNG1	NPC2	QSOX1	SH2D1A	XRCC5
C4BPA	DDAH1	GUK1	KPRP	NPEPPS	QSOX2	SHBG	XRCC6
C4orf48	DDX3X	H1-3	KRT1	NPM1	RAB10	SHMT2	XXYLT1
C5	DDX5	H1-4	KRT10	NPTN	RAB11B	SIL1	YBX1

C6	DEFA1	H1-5	KRT13	NPTX1	RAB14	SIRPA	YBX3
C7	DHX9	H2AC11	KRT14	NPTXR	RAB1A	SKP1	YWHAB
C8A	DIAPH1	H2AC4	KRT16	NRAS	RAB1B	SLC12A2	YWHAE
C8B	DKK3	H2AZ1	KRT17	NRCAM	RAB2A	SLC1A2	YWHAG
C8G	DLAT	H2BC11	KRT2	NRP2	RAB3A	SLC1A3	YWHAH
C9	DLD	H3C1	KRT31	NRXN1	RAB5A	SLC25A12	YWHAQ
CA1	DLG4	H4-16	KRT33A	NRXN2	RAB5C	SLC25A18	YWHAZ
CA10	DLST	HABP2	KRT33B	NRXN3	RAB6B	SLC25A3	ZAP70
CA14	DMBT1	HADH	KRT4	NSF	RAB7A	SLC25A4	ZBTB40
CA2	DNAJA1	HBA1	KRT5	NT5DC1	RAB8B	SLC39A12	ZG16B
CACHD1	DNAJB1	HBB	KRT6A	NTM	RAC1	SLC3A2	ZNF518A
CACNA2D1	DNAJC3	HBD	KRT6B	NUCB1	RACK1	SLC5A2	

Supplementary Table S6. Final Serum Proteome List. List of Serum exosomal gene names obtained from overlap of the EVpedia, EXOCARTA and Vesiclepedia. Exosome gene names mined from the literature were also included.

**Proteome of Serum-derived exosomes - Gene name**							
A1BG	CALML5	EEF2	H2BC13	ITIH2	MUC19	PTPRJ	STOM
A2M	CAP1	EFEMP1	H2BC14	ITIH3	MUC5B	PTPRR	STX5
AASS	CARF	EFNA5	H2BC15	ITIH4	MVP	PTPRZ1	STX7
ABCA7	CASP14	EGFR	H2BFS	ITM2A	MXD3	PTTG1IP	STXBP2
ABCA9	CAST	EIF2B5	H3-3A	ITM2B	MYBPC2	PTX3	SVEP1
ABCB1	CAT	EIF4A1	H3C1	JAKMIP3	MYCT1	PUDP	SYBU
ABCB4	CAVIN1	EIF4A2	H3F3C	JCHAIN	MYH10	PXDN	SYNCRIP
ABCC2	CAVIN2	ELMO1	H4-16	JUP	MYH11	PZP	SYNE1
ABCG2	CCAR2	ELMO2	HABP2	KANSL1	MYH13	QSOX1	SYNE2
ABHD17B	CCDC114	EMILIN1	HBA1	KATNAL2	MYH14	R3HDM2	SYTL3
ABL2	CCDC150	EMILIN2	HBB	KCMF1	MYH2	RAB10	TAF4
ACAA2	CCDC158	EML4	HBD	KCNAB2	MYH9	RAB1A	TAF6
ACAP1	CCDC159	ENO1	HBE1	KCND3	MYL12A	RAB1B	TAGLN2
ACAP2	CCDC17	ENO2	HBG1	KCTD12	MYL12B	RAB1C	TALDO1
ACOT9	CCDC183	ENO3	HBG2	KDM5C	MYL3	RAB27A	TBC1D1
ACTA1	CCDC39	ENPEP	HBS1L	KIAA0825	MYL4	RAB3A	TBC1D23
ACTA2	CCDC40	ENTPD5	HCFC2	KIAA1586	MYL6	RAB3B	TBL1X
ACTB	CCDC63	EP300	HDGFL3	KIF16B	MYL6B	RAB3C	TBL1Y
ACTBL2	CCDC74A	EPB41	HECTD4	KIF20B	MYL9	RAB3D	TBX1
ACTC1	CCDC74B	EPC1	HES6	KIF3B	MYO18A	RAB5A	TBX10
ACTG1	CCDC80	EPCAM	HGFAC	KIF7	MYO18B	RAB5B	TCP1
ACTG2	CCDC81	EPHB3	HIST1H1T	KLHL1	MYO19	RAB7A	TDP1
ACTN1	CCP110	EPN2	HIST1H2AA	KLHL41	MYO1F	RACK1	TENT2
ACTN2	CCT2	EPPK1	HIST1H2AC	KLK3	MYO1G	RAD54B	TESPA1
ACTN3	CCT3	EPS8	HIST1H2AH	KLKB1	MYSM1	RADX	TEX15
ACTN4	CCT4	EPX	HIST1H2BB	KNG1	NACA	RALGAPB	TF

ACTR3	CCT5	ERAL1	HIST1H2BD	KRT1	NACA2	RAP1A	TFIP11
ADAM10	CCT6A	ERAP2	HIST1H2BH	KRT10	NACC2	RAP1B	TFPI
ADGRV1	CCT6B	ERBB2	HIST1H2BO	KRT14	NAGPA	RAP2B	TFRC
ADIPOQ	CCT7	ERC1	HIST2H2AA3	KRT15	NAT16	RASSF8	TGFB1
AFM	CCT8	ERVK-5	HIST2H2AB	KRT16	NCL	RBM17	TGFBI
AGRP	CD14	ESYT1	HIST2H2AC	KRT17	NDC80	RBMX2	TGM2
AGT	CD207	ETAA1	HIST2H2BE	KRT2	NDUFS1	RBP4	THBS1
AHSA2P	CD24	EVC	HIST2H2BF	KRT20	NF1	RELN	THBS3
AHSG	CD276	EVPL	HIST2H3A	KRT222	NF2	RGMB	THBS4
AKT1	CD36	EXTL2	HIST3H2A	KRT23	NFE2L3	RGPD1	THEG
AKT2	CD59	EYS	HIST3H2BB	KRT24	NID1	RGPD2	TIGD2
AKT3	CD5L	EZR	HIST3H3	KRT25	NIN	RGPD5	TIGD6
ALB	CD63	F10	HLA-A	KRT26	NKX3-2	RGPD8	TJAP1
ALDH16A1	CD81	F11	HLA-B	KRT27	NLRP1	RHOA	TJP2
ALDOA	CD9	F12	HLA-C	KRT28	NPAS3	RIC8A	TLN1
ALG11	CDC25C	F13A1	HMBS	KRT3	NPPB	RIT1	TLN2
ALKBH5	CDC27	F13B	HMCN1	KRT31	NPPC	RMI1	TLR4
ALPI	CDHR2	F2	HMGB1	KRT32	NRAP	RNF166	TLR6
ALPL	CDKN2AIPNL	F5	HMGB1P1	KRT33A	NSDHL	RNF169	TMC2
AMBP	CDR2L	F7	HMGN1	KRT33B	NSF	RNF19B	TMCO3
AMPD3	CELSR3	F9	HNRNPC	KRT34	NUMBL	RNF25	TMEM181
ANGPT1	CEMIP	FABP5	HNRNPH1	KRT35	NUP58	RNH1	TMEM63A
ANGPTL1	CENPE	FAM161A	HNRNPH2	KRT36	OASL	ROBO4	TNC
ANGPTL3	CENPF	FAM161B	HNRNPK	KRT37	OBSCN	RPL13	TNRC6A
ANGPTL6	CENPJ	FAM171A1	HNRNPU	KRT38	ODF2L	RPL13A	TNXB
ANK1	CEP126	FAM25A	HOOK2	KRT40	OIT3	RPL18	TOLLIP
ANKMY1	CEP164	FAM78B	HP	KRT5	OLFM4	RPL29	TOP2A
ANKRD65	CES2	FAM81A	HPR	KRT6A	OR2T6	RPLP2	TOP3B
ANO3	CFAP73	FASN	HPRT1	KRT6B	OR4C16	RPS16	TP53BP2
ANO6	CFB	FBLN1	HPX	KRT6C	OR4K14	RPS27A	TPI1
ANPEP	CFD	FBP1	HRG	KRT71	ORM1	RPS28	TPM1
ANXA1	CFH	FBXO22	HSP90AA1	KRT72	ORM2	RPSA	TPM2
ANXA11	CFHR1	FBXO28	HSP90AA2P	KRT73	OS9	RPUSD3	TPM3
ANXA2	CFHR2	FCGBP	HSP90AA4P	KRT75	OSBPL8	RSU1	TPM4
ANXA2P2	CFHR5	FCHSD1	HSP90AB1	KRT76	OTOA	RYR2	TPP2
ANXA3	CFI	FCN1	HSP90AB2P	KRT77	OTOF	S100A11	TPR
ANXA4	CFL1	FCN2	HSP90AB3P	KRT78	OTUD4	S100A14	TPT1
ANXA5	CFL2	FCN3	HSP90B1	KRT79	OTUD7A	S100A7	TRAP1
ANXA6	CFP	FERMT3	HSPA1A	KRT80	P4HB	S100A7A	TREX2
ANXA7	CFTR	FGA	HSPA1L	KRT81	PABPC1	S100A8	TRIM24
ANXA8	CHD3	FGB	HSPA2	KRT82	PABPC4	S100A9	TRIM28
ANXA8L1	CHD7	FGG	HSPA4L	KRT83	PADI3	S100P	TRIM63

AP1AR	CKAP4	FGL1	HSPA5	KRT84	PARN	SAA1	TRIM7
AP3M1	CKMT1A	FHIT	HSPA6	KRT85	PARP9	SAA2	TRMT11
APCS	CLASP2	FLG	HSPA7	KRT86	PARPBP	SAA4	TRPM6
APEH	CLCA1	FLNA	HSPA8	KRT87P	PARVB	SACS	TRPS1
APLP2	CLCN4	FLNB	HSPB1	KRT9	PASD1	SAP25	TSG101
APOA1	CLCN6	FLNC	HSPG2	L1CAM	PCCA	SART1	TSNAXIP1
APOA2	CLCNKA	FLOT1	HUNK	LAMA4	PCDHB3	SATL1	TSPAN14
APOA4	CLCNKB	FLRT1	HUWE1	LAMB1	PCDHGA1	SBF1	TSPAN15
APOA5	CLEC3B	FN1	HYOU1	LAMB4	PCDHGA10	SBSN	TSPAN33
APOB	CLIC1	FOLH1	IDE	LAMC1	PCYOX1	SCAMP1	TSPAN9
APOC1	CLTA	FOXP4	IFT122	LAMP1	PDCD6	SCFD1	TTBK1
APOC2	CLTC	FREM3	IGFALS	LAMP2	PDCD6IP	SCIN	TTC37
APOC3	CLTCL1	FTH1	IGHA1	LARGE1	PDE12	SCNN1D	TTN
APOD	CLU	FTL	IGHA2	LATS2	PDE6B	SCUBE3	TTR
APOE	CMYA5	FYCO1	IGHD	LBP	PDF	SDCBP	TTYH3
APOF	CNDP1	GANAB	IGHE	LCA5	PDLIM1	SEC24C	TUBA1A
APOH	COL18A1	GAPDH	IGHG1	LCN2	PDLIM5	SEL1L	TUBA1B
APOL1	COL1A2	GAS1	IGHG2	LCP2	PECAM1	SELENOP	TUBA1C
APOM	COL22A1	GATA3	IGHG3	LDHA	PEF1	SELP	TUBA3C
ARAP1	COL2A1	GC	IGHG4	LDHAL6A	PEG10	SEMA6D	TUBA3E
ARCN1	COL6A1	GCFC2	IGHM	LDHB	PEX1	SEMG1	TUBA4A
ARF1	COL6A2	GGCT	IGHMBP2	LDHC	PEX5L	SERPINA1	TUBA4B
ARF3	COL6A3	GGT1	IGHV1-2	LGALS3BP	PF4	SERPINA3	TUBA8
ARF5	COLEC10	GGT2	IGHV1-46	LGALS7	PF4V1	SERPINA4	TUBB
ARFGEF1	COLEC11	GGT3P	IGHV1-69	LGALS9	PFN1	SERPINA5	TUBB1
ARHGDIA	COLGALT2	GGT7	IGHV2-5	LGALS9B	PGAM1	SERPINA6	TUBB2A
ARHGEF18	COMMD3	GGTLC2	IGHV2-70	LGALS9C	PGK1	SERPINA7	TUBB2B
ARMC10	COQ4	GGTLC3	IGHV3-11	LGI3	PGLYRP2	SERPINB3	TUBB3
ARNT	COQ8B	GIPC1	IGHV3-13	LIM2	PHACTR1	SERPINB4	TUBB4A
ARPC3	CORO1A	GJD4	IGHV3-23	LIMS1	PIGR	SERPINB5	TUBB4B
ASPRV1	CORO1C	GLA	IGHV3-30	LIMS2	PIK3CD	SERPINC1	TUBB6
ASXL1	CORO6	GLG1	IGHV3-33	LMNA	PIP4K2C	SERPIND1	TUBB8
ATAD2	CP	GLIPR2	IGHV3-48	LMNB1	PIWIL2	SERPINF1	TUBB8B
ATF6	CPA5	GLMN	IGHV3-53	LPA	PKD1	SERPINF2	TXLNA
ATG2B	CPB2	GLUL	IGHV3-7	LPAL2	PKD1L3	SERPING1	TXN
ATP13A3	CPN1	GNA12	IGHV3-9	LRG1	PKHD1L1	SETD1A	UBA1
ATP1A1	CPN2	GNA13	IGHV4-34	LRIF1	PKM	SETD2	UBA52
ATP23	CPNE1	GNAI1	IGHV4-39	LRP1	PLAT	SEZ6L2	UBB
ATP2B4	CPNE2	GNAI2	IGHV4-59	LRP1B	PLEC	SF3B2	UBC
ATP5F1A	CPNE3	GNAI3	IGKC	LRPPRC	PLEK	SFN	UBTFL6
ATP5F1B	CPNE4	GNAL	IGKV1-16	LRRC37A	PLEKHA4	SFPQ	UGGT1
ATRN	CPNE5	GNAO1	IGKV1-17	LRRC37A2	PLEKHA7	SFTPA1	UGGT2

ATXN2L	CPNE6	GNAS	IGKV1-33	LRRC37A3	PLEKHS1	SFTPA2	UGP2
AZGP1	CPNE7	GNAT1	IGKV1-39	LRRC45	PLG	SFTPB	UHRF1BP1
B2M	CPNE8	GNAT2	IGKV1-5	LRRC71	PLGLB1	SGCG	UPF3A
B4GALT6	CPNE9	GNAT3	IGKV1D-12	LRRC8D	PLTP	SH3BP2	UQCRC1
BAIAP2	CROCC	GNB1	IGKV1D-16	LRRFIP2	PLXNB3	SH3GL2	UROC1
BAIAP2L1	CRP	GNB1L	IGKV1D-33	LRRIQ4	POF1B	SHANK1	USP1
BANF1	CSPG5	GNB2	IGKV1D-39	LTBP1	POLRMT	SHBG	USP29
BAP1	CSTB	GNB3	IGKV2-30	LTF	PON1	SIGLEC1	USP33
BASP1	CTSG	GNB4	IGKV2D-28	LUM	POTEE	SIPA1L1	USP9X
BBOF1	CTTNBP2	GOLGA3	IGKV2D-40	LYPD3	POTEF	SIPA1L2	USP9Y
BCHE	CUL4B	GOLGA4	IGKV3-11	LYRM4	POTEI	SLC1A5	UTRN
BCL11A	CUX1	GOLGA6L1	IGKV3-15	LYZ	POTEJ	SLC27A4	VARS
BEND6	CYFIP2	GOLGA6L2	IGKV3-20	MACF1	POTEKP	SLC2A1	VASP
BGN	DAAM1	GOLGA6L6	IGKV4-1	MAD1L1	POU3F4	SLC2A3	VCL
BHMT	DBH	GOLGA8CP	IGKV5-2	MAFB	PPBP	SLC36A4	VCP
BIRC2	DCC	GOLGA8M	IGLC1	MAGEB1	PPIA	SLC3A2	VIL1
BLMH	DCD	GOLGA8N	IGLC2	MAGED4	PPL	SLC43A1	VIM
BLVRB	DCP1B	GOLGA8O	IGLC6	MAGEL2	PPP1R9A	SLC4A1	VTN
BMP2K	DCTN1	GOLGA8R	IGLC7	MAGI2	PPP2R3A	SLC4A9	VWF
BMS1	DCTN2	GOLGB1	IGLL1	MAMDC2	PPRC1	SLC7A5	WASL
BNIP5	DDX17	GOSR2	IGLL5	MAPK7	PRDM14	SLF2	WDR1
BSG	DDX49	GP1BB	IGLV1-40	MAPK8IP1	PRDX1	SLPI	WDR19
BST2	DEFA1	GP9	IGLV1-44	MAPKAPK5-AS1	PRDX2	SMC1A	WDR3
C16orf89	DEFA3	GPD1	IGLV1-47	MAPRE2	PRDX6	SMC5	WDR5
C19orf57	DERA	GPD2	IGLV1-51	MARCH6	PRG4	SMG5	WDR81
C1orf105	DES	GPR156	IGLV2-11	MARS	PRKCH	SNAP25	WNT1
C1QA	DGCR6	GPR33	IGLV2-14	MASP1	PRKDC	SNPH	XPO7
C1QB	DGKH	GPX3	IGLV2-23	MASP2	PRMT9	SNX30	YWHAB
C1QC	DHRS3	GRID1	IGLV3-1	MAST2	PROS1	SOD1	YWHAE
C1QTNF3	DHX29	GSDMA	IGLV3-19	MATR3	PRPF31	SOGA1	YWHAG
C1R	DHX57	GSN	IGLV3-21	MBL2	PRPF39	SORCS3	YWHAH
C1RL	DHX9	GSTP1	IGLV3-25	MDN1	PRRC2C	SP2	YWHAQ
C1S	DMBT1	GSTT1	IGLV6-57	MECP2	PRSS1	SP4	YWHAZ
C2	DMKN	GTF2H4	IGLV7-43	MED10	PRSS3	SPATA32	ZBTB37
C3	DNA2	GTF2IRD2	IKBIP	MEFV	PRSS3P2	SPEF2	ZBTB41
C4A	DNAH5	GTF2IRD2B	IL12RB1	MEI1	PSMA1	SPG7	ZFPM2
C4B	DNAH8	GXYLT2	IL1R1	MFN1	PSMA2	SPICE1	ZFYVE1
C4BPA	DNAJC18	GYPA	IL27RA	MGAM	PSMA3	SPOCD1	ZFYVE19
C4BPB	DOCK10	GZMH	IL36G	MGAT5	PSMA4	SPP2	ZGPAT
C5	DPH1	H1-1	ILK	MIPEP	PSMA5	SPRR1B	ZNF101
C6	DPP4	H1-2	INSM2	MME	PSMA6	SPSB4	ZNF169
C6orf132	DRP2	H1-3	INTS6	MMP27	PSMA7	SPTA1	ZNF232

C7	DSG1	H1-4	IPO5	MMRN1	PSMA8	SPTB	ZNF281
C8A	DSG2	H2AC11	IQGAP1	MOCS3	PSMB1	SPTBN2	ZNF292
C8B	DSP	H2AC14	ISYNA1	MPO	PSMB10	SPTBN4	ZNF318
C8G	DST	H2AC4	ITGA2	MRC1	PSMB2	SPTBN5	ZNF43
C9	DYNLL1	H2AC7	ITGA2B	MRE11	PSMB3	SQOR	ZNF518A
C9orf43	DYNLL2	H2AFJ	ITGA3	MROH5	PSMB4	SRI	ZNF644
CA1	E2F1	H2AFV	ITGA5	MRPS35	PSMB5	SRPX2	ZNF776
CA2	ECM1	H2AFX	ITGA6	MSN	PSMB6	ST13	ZSCAN12
CAB39L	EEF1A1	H2AFY	ITGAM	MST1	PSMB7	ST13P4	ZWINT
CABCOCO1	EEF1A1P5	H2AFY2	ITGB1	MTFR2	PSMB8	ST13P5	
CAD	EEF1A2	H2AZ1	ITGB2	MTMR2	PSMB9	ST8SIA4	
CADPS2	EEF1B2	H2BC10	ITGB3	MTPN	PTAFR	STAT2	
CALM1	EEF1D	H2BC11	ITGB6	MTUS1	PTCH2	STEAP3	
CALML3	EEF1G	H2BC12	ITIH1	MUC16	PTGFRN	STK36	

Supplementary Table S7. Final Plasma Proteome List. List of Plasma exosomal gene names obtained from the overlap of the EVpedia, EXOCARTA and Vesiclepedia. Exosome gene names mined from the literature were also included.

**Proteome of Plasma-derived exosomes - Gene name**							
A1BG	CA3	DSP	HIST1H2AA	IGLC7	LIPC	PIPOX	SNTN
A2M	CABIN1	DUS3L	HIST1H2AB	IGLL1	LOC100132941	PKM	SOHLH1
A2ML1	CALML3	DYNC2H1	HIST1H2AC	IGLL5	LOC100134256	PLCD3	SPAG7
ABCA7	CALML5	EBF2	HIST1H2AD	IGLV10-54	LOC100134397	PLG	SPATA2L
ACACA	CAND1	EBP	HIST1H2AE	IGLV1-36	LOC100289290	PLTP	SPECC1
ACSM1	CAPN1	ECM1	HIST1H2AG	IGLV1-44	LOC100291786	PLXNA4	SPO11
ACTA1	CAPN2	EEF1A1	HIST1H2AH	IGLV1-47	LOC100291917	PNKD	SPTBN5
ACTA2	CAPZA2	EEF1A2	HIST1H2AI	IGLV1-51	LOC100293211	PNKP	SRGAP2
ACTB	CASC5	EFEMP1	HIST1H2AJ	IGLV2-11	LOC100293534	POLQ	SRGAP2C
ACTBL2	CASP8AP2	EIF2A	HIST1H2AK	IGLV2-14	LOC100652743	POMGNT1	ST6GALNAC2
ACTC1	CAT	EIF3C	HIST1H2AL	IGLV2-18	LOC100652818	PON1	STOM
ACTG1	CAV1	ENO1	HIST1H2AM	IGLV2-8	LOC100653084	PON3	STX7
ACTG2	CCDC154	ENO2	HIST2H2AA3	IGLV3-10	LOC100653210	POTEE	STXBP1
ACTN1	CCDC171	ENPP1	HIST2H2AA4	IGLV3-19	LOC642131	POTEF	SUPT6H
ACTN3	CCDC30	ENPP5	HIST2H2AB	IGLV3-21	LPA	POTEI	SVEP1
ACTN4	CCDC37	EPHA1	HIST2H2AC	IGLV3-25	LRG1	POTEJ	SYN1
ACTR5	CCDC80	EPX	HIST3H2A	IGLV3-27	LRP1	POTEKP	SYNE1
ADAL	CCHCR1	EXOC4	HJURP	IGLV4-3	LRP1B	POTEM	SYNJ1
ADAMTS16	CCPG1	EXOSC1	HLA-A	IGLV4-69	LTBP1	PPARG	SYNPO
ADAMTS9	CD163L1	F10	HLA-B	IGLV7-43	LTBP2	PPIA	SYP
ADAR	CD274	F11	HLA-DRA	IGLV7-46	LUM	PPIG	SYT2
ADCY7	CD46	F11R	HLA-DRB5	IHH	LY75	PPP1R12C	TADA3
ADIPOQ	CD55	F12	HLA-E	IK	LY75-CD302	PPT2	TBX20

AFM	CD59	F13A1	HP	IL1B	LYZ	PRDX2	TDRD1
AFMID	CD5L	F13B	HPR	IL1RAP	MACROD1	PREB	TET1
AGT	CD63	F2	HPX	IL1RAPL2	MANF	PREPL	TF
AHSG	CD70	F5	HRG	IL26	MAP1LC3A	PRG4	TFRC
ALB	CD81	F9	HRNR	IL6	MAP4	PROC	TGFB1
ALPP	CD9	FABP5	HSP72*	ILK	MAP7D3	PROM2	TGFBI
AMBP	CD93	FAM150A	HSPA1A	INCENP	MAPT	PROS1	THBS1
AMZ1	CDH5	FAM196B	HSPA1B	INTS11	MASP1	PROZ	THEMIS
ANGPTL4	CENPH	FAM207A	HSPA1L	IQCF6	MASP2	PRPF31	TLK2
ANK2	CEP135	FAM208B	HSPA2	Irs1	MBD5	PRRC2A	TLN1
ANKAR	CETP	FAM48A	HSPA5	ITGA2B	MBL2	PRSS1	TLN2
ANKDD1B	CFB	FAM78B	HSPA6	ITGB3	MDN1	PRSS3	TMEM131
ANKLE1	CFB BF	FASLG	HSPA7	ITIH1	MEX3B	PSEN1	TMEM132C
ANKRD12	CFD	FBF1	HSPA8	ITIH2	MFNG	PSENEN	TMEM151A
ANKRD26	CFH	FBLN1	HSPB1	ITIH3	MIF	PSMB3	TMEM201
ANKUB1	CFHR1	FCGBP	HTR2A	ITIH4	MKL1	PSMC2	TMEM232
ANXA5	CFHR2	FCN1	IDO2	JARID2	MMRN1	PSMC5	TMF1
AP2A2	CFHR3	FCN2	IFI30	JCHAIN	MOG	PTF1A	TMPRSS13
APCS	CFHR5	FCN3	IGF1	JMY	MRE11A	PTPRM	TNF
APH1A	CFI	FGA	IGF2	KATNB1	MRPL32	PZP	TP63
APOA1	CFL1	FGB	IGFALS	KCND2	MSH6	RAB10	TPD52L2
APOA2	CFP	FGF13	IGFBP3	KCNQ5	MSN	RAB3GAP1	TPM3
APOA4	CHD4	FGF2	IGHA1	KDM2B	MST1	RAB5B	TPM4
APOB	CHD9	FGFR1OP	IGHA2	KDM4D	MYBL1	RAB6A	TRAJ56
APOC1	CHRNG	FGFRL1	IGHD	KDM5A	MYH1	RAB8B	TREML4
APOC2	CHST11	FGG	IGHG1	KIAA0586	MYH13	RALGAPA2	TRIM32
APOC3	CHSY3	FHAD1	IGHG2	KIAA1161	MYH2	RARS	TRIM65
APOC4	CIB2	FIGNL2	IGHG3	KIF13A	MYH3	RBM23	TRIOBP
APOD	CILP	FLG2	IGHG4	KIF19	MYH4	RBP4	TRIP11
APOE	CLDN4	FLNA	IGHM	KIRREL	MYH6	REG1A	TROAP
APOF	CLEC3B	FLNB	IGHV1-18	KLC2	MYH7	RELN	TRPC5
APOH	CLIC1	FLOT1	IGHV1-2	KLKB1	MYH8	REST	TSG101
APOL1	CLNK	FLOT2	IGHV1-45	KLRF1	MYH9	RFC4	TSGA13
APOM	CLTC	FN1	IGHV1-46	KNG1	MYO15A	RFX8	TTC12
APOPT1	CLTCL1	FRMD3	IGHV1-8	KRT1	MYO3A	RMND1	TTN
APP	CLU	G6PC2	IGHV2-70	KRT10	MYO6	RNF111	TTR
APPL2	CMBL	GAK	IGHV3-13	KRT12	NAE1	RNF17	TUBB
ARG1	CNDP1	GAP43	IGHV3-15	KRT13	NCAM1	RNF213	TUBB1
ARHGAP15	CNOT6L	GAPDH	IGHV3-20	KRT14	NCL	RNF219	TUBB2A
ARHGAP8	COL25A1	GC	IGHV3-23	KRT15	NCOA2	RPGRIP1L	TUBB2B
ARHGEF11	COLEC10	GCLM	IGHV3-30	KRT16	NCSTN	RPL29	TUBB3
ASPM	COMP	GCN1L1	IGHV3-33	KRT17	NEB	RPL37	TUBB4A

ATG2B	CP	GDNF	IGHV3-38	KRT18	NEDD4	RPS27A	TUBB4B
ATP1A1	CPB2	Gfap	IGHV3-48	KRT19	NEFL	RPS6KA4	TUBB6
ATP1A2	CPN1	GJB1	IGHV3-53	KRT2	NHS	RTKN	TUBB8
ATP1A3	CPN2	GLRA2	IGHV3-7	KRT24	NIN	RYR2	TUBGCP6
ATP2A3	CR1	GLUL	IGHV3-72	KRT25	NIPBL	S100A7	TXNRD3
ATP6AP2	CROCC	GMEB1	IGHV3-73	KRT26	NLGN1	S100A8	UBA52
ATR	CRTAC1	GOLGA3	IGHV3-9	KRT27	NOA1	S100A9	UBB
ATRN	CSPG4	GOT1	IGHV3OR16-10	KRT28	Nptx2	SAA1	UBC
AZGP1	CSRNP1	GP1BA	IGHV3OR16-12	KRT31	Nrgn	SAA2	UBQLNL
B2M	CSTA	GP1BB	IGHV3OR16-9	KRT32	NRXN2	SAA4	UCKL1
B4GALT7	CSTB	GPLD1	IGHV4-34	KRT33B	NRXN3	SAMD8	UGDH
BACE1	CTBS	GPR87	IGHV4-39	KRT35	NSD1	SASH1	USP9X
BCAM	CTSD	GPX3	IGHV4-4	KRT37	NTN5	SCGB1A1	UTRN
BCHE	CUL9	GPX4	IGHV5-51	KRT4	NUDT15	SCUBE3	VASP
BEND4	CWC25	GRIA4	IGHV6-1	KRT5	OAZ3	SDCBP	VCL
BGLAP	CYP1A2	GRID2	IGJ	KRT6A	OBSCN	SELENOP	VCP
BLM	CYP51A1	GRIN1	IGKC	KRT6B	OCEL1	SELL	VPS13C
BRCA2	DAPL1	GRIPAP1	IGKV1-16	KRT6C	ODF2	SEPTIN8	VTN
BRPF1	DBI	GSDMA	IGKV1-17	KRT7	OLFM2	SERPINA1	VWF
BTD	DCAF6	GSN	IGKV1-33	KRT75	OPN4	SERPINA10	WAC
C19orf68	DCD	GSS	IGKV1-39	KRT76	OR4K1	SERPINA3	WDR1
C1orf174	DCLRE1B	GYPA	IGKV1-5	KRT77	ORM1	SERPINA4	WDR35
C1orf228	DCSTAMP	H2AFJ	IGKV1-6	KRT79	ORM2	SERPINA5	WDR43
C1QA	DCX	H2AFV	IGKV1-8	KRT8	PALB2	SERPINA6	WFDC3
C1QB	DDX21	H2AFX	IGKV1D-12	KRT80	PARD3B	SERPINA7	YIPF1
C1QC	DDX51	H2AFZ	IGKV1D-13	KRT81	PARP4	SERPINB9	YWHAH
C1R	DEFA3	H2BC10	IGKV1D-33	KRT83	PCDH8	SERPINC1	YWHAZ
C1RL	DENND2D	H4-16	IGKV2-30	KRT85	PCLO	SERPIND1	ZBTB38
C1S	DES	HABP2	IGKV2D-24	KRT86	PCYOX1	SERPINF1	ZC3HC1
C2	DGAT1	HBA1	IGKV2D-28	KRT9	PDCD6IP	SERPINF2	ZFHX4
C3	DGCR14	HBA2	IGKV2D-29	L1CAM	PDGFRA	SERPING1	ZFP64
C4A	DHX30	HBB	IGKV2D-40	LAMC1	PEAK1	SETD1A	ZNF132
C4B	DIP2B	HBD	IGKV3-11	Lamp1	PECAM1	SH3BGRL3	ZNF14
C4BPA	DMXL1	HBE1	IGKV3-15	LAMP2	PEG10	SHANK1	ZNF177
C4BPB	DNAH2	HBG1	IGKV3-20	LBP	PER3	SHANK3	ZNF215
C5	DNAH3	HBG2	IGKV3-7	LCAT	PF4	SHBG	ZNF286B
C6	DNAH5	HCN3	IGKV3D-11	LDB1	PFDN6	SHROOM3	ZNF345
C7	DNAH7	HERC1	IGKV3D-20	LGALS3BP	PFN1	SLC1A3	ZNF532
C8A	DNAJC7	HES1	IGKV4-1	LGALS7	PGLYRP2	SLC2A1	ZNF561
C8B	DNPEP	HGF	IGKV6-21	LGALS7B	PIGR	SLC44A1	ZNF624
C8G	DOPEY1	HGFAC	IGKV6D-41	LHFPL3	PIGS	SLC4A1	ZNF74
C9	DSCAM	HGS	IGLC1	LILRA1	PIK3C2A	SLPI	

C9orf78	DSG1	HIP1	IGLC2	LIMS1	PION	SMO	

References

1. Goetzl, E.J.; Nogueras-Ortiz, C.; Mustapic, M.; Mullins, R.J.; Abner, E.L.; Schwartz, J.B.; Kapogiannis, D. Deficient neurotrophic factors of CSPG4-type neural cell exosomes in Alzheimer disease. *FASEB J.* **2019**, *33*, 231–238, doi:10.1096/fj.201801001.
2. Goetzl, E.J.; Abner, E.L.; Jicha, G.A.; Kapogiannis, D.; Schwartz, J.B. Declining levels of functionally specialized synaptic proteins in plasma neuronal exosomes with progression of Alzheimer's disease. *FASEB J.* **2018**, *32*, 888–893, doi:10.1096/fj.201700731r.
3. Mettenburg, J.M.; Webb, D.J.; Gonias, S.L. Distinct binding sites in the structure of α2-macroglobulin mediate the interaction with β-amyloid peptide and growth factors. *J. Biol. Chem.* **2002**, *277*, 13338–13345, doi:10.1074/jbc.m106792200.
4. Narita, M.; Holtzman, D.M.; Schwartz, A.L.; Bu, G. α2-macroglobulin complexes with and mediates the endocytosis of β-amyloid peptide via cell surface low-density lipoprotein receptor-related protein. *J. Neurochem.* **2002**, *69*, 1904–1911, doi:10.1046/j.1471-4159.1997.69051904.x.
5. Guo, C.; Zhou, H.-X. Fatty acids compete with aβ in binding to serum albumin by quenching its conformational flexibility. *Biophys. J.* **2019**, *116*, 248–257, doi:10.1016/j.bpj.2018.11.3133.
6. Milojevic, J.; Costa, M.; Ortiz, A.M.; Jorquera, J.I.; Melacini, G. In Vitro amyloid-β binding and inhibition of amyloid-β self-association by therapeutic albumin. *J. Alzheimer's Dis.* **2013**, *38*, 753–765, doi:10.3233/jad-131169.
7. Picón-Pagès, P.; Bonet, J.; García-García, J.; Garcia-Buendia, J.; Gutierrez, D.; Valle, J.; Gómez-Casuso, C.E.; Sidelkivska, V.; Alvarez, A.; Perálvarez-Marín, A.; et al. Human albumin impairs amyloid β-peptide fibrillation through its c-terminus: from docking modeling to protection against neurotoxicity in Alzheimer's disease. *Comput. Struct. Biotechnol. J.* **2019**, *17*, 963–971, doi:10.1016/j.csbj.2019.06.017.
8. Calero, M.; Rostagno, A.; Ghiso, J. Search for amyloid-binding proteins by affinity chromatography. *Methods Mol. Biol.* **2012**, *849*, 213–223, doi:10.1007/978-1-61779-551-0_15.
9. Liko, I.; Mák, M.; Klement, E.; Hunyadi-Gulyas, E.; Pázmány, T.; Medzihradszky, K.F.; Urbányi, Z. Evidence for an extended interacting surface between β-amyloid and serum amyloid P component. *Neurosci. Lett.* **2007**, *412*, 51–55, doi:10.1016/j.neulet.2006.10.052.
10. Mold, M.; Shrive, A.K.; Exley, C. Serum amyloid p component accelerates the formation and enhances the stability of amyloid fibrils in a physiologically significant under-saturated solution of amyloid-β42. *J. Alzheimer's Dis.* **2012**, *29*, 875–881, doi:10.3233/jad-2012-120076.
11. Paula-Lima, A.C.; Tricerri, M.A.; Brito-Moreira, J.; Bomfim, T.R.; Oliveira, F.F.; Magdesian, M.H.; Grinberg, L.T.; Panizzutti, R.; Ferreira, S.T. Human apolipoprotein A–I binds amyloid-β and prevents Aβ-induced neurotoxicity. *Int. J. Biochem. Cell Biol.* **2009**, *41*, 1361–1370, doi:10.1016/j.biocel.2008.12.003.
12. Koldamova, R.P.; Lefterov, I.M.; Lefterova, M.I.; Lazo, J.S. Apolipoprotein A-I directly interacts with amyloid precursor protein and inhibits aβ aggregation and toxicity. *Biochemistry* **2001**, *40*, 3553–3560, doi:10.1021/bi002186k.
13. Shih, Y.-H.; Tsai, K.-J.; Lee, C.-W.; Shiesh, S.-C.; Chen, W.-T.; Pai, M.-C.; Kuo, Y.-M. Apolipoprotein C-III is an amyloid-β-binding protein and an early marker for Alzheimer's disease. *J. Alzheimer's Dis.* **2014**, *41*, 855–865, doi:10.3233/jad-140111.
14. Koudinov, A.R.; Berezov, T.T.; Kumar, A.; Koudinova, N.V. Alzheimer's amyloid β interaction with normal human plasma high density lipoprotein: Association with apolipoprotein and lipids. *Clin. Chim. Acta* **1998**, *270*, 75–84, doi:10.1016/s0009-8981(97)00207-6.
15. Cui, Y.; Huang, M.; He, Y.; Zhang, S.; Luo, Y. Genetic ablation of apolipoprotein A-IV Accelerates Alzheimer's disease pathogenesis in a mouse model. *Am. J. Pathol.* **2011**, *178*, 1298–1308, doi:10.1016/j.ajpath.2010.11.057.
16. Abildayeva, K.; Berbée, J.F.P.; Blokland, A.; Jansen, P.J.; Hoek, F.J.; Meijer, O.; Lütjohann, D.; Gautier, T.; Pillot, T.; De Vente, J.; et al. Human apolipoprotein C-I expression in mice impairs learning and memory functions. *J. Lipid Res.* **2008**, *49*, 856–869, doi:10.1194/jlr.m700518-jlr200.
17. Ghosh, S.; Sil, T.B.; Dolai, S.; Garai, K. High-affinity multivalent interactions between apolipoprotein E and the oligomers of amyloid-β. *FEBS J.* **2019**, *286*, 4737–4753, doi:10.1111/febs.14988.
18. Garai, K.; Verghese, P.B.; Baban, B.; Holtzman, D.M.; Frieden, C. The binding of apolipoprotein e to oligomers and fibrils of amyloid-β alters the kinetics of amyloid aggregation. *Biochemistry* **2014**, *53*, 6323–6331, doi:10.1021/bi5008172.
19. Darvesh, S. Butyrylcholinesterase as a diagnostic and therapeutic target for Alzheimer's disease. *Curr. Alzheimer Res.* **2016**, *13*, 1173–1177, doi:10.2174/1567205013666160404120542.
20. Diamant, S.; Podoly, E.; Friedler, A.; Ligumsky, H.; Livnah, O.; Soreq, H. Butyrylcholinesterase attenuates amyloid fibril formation in vitro. *Proc. Natl. Acad. Sci. USA* **2006**, *103*, 8628–8633, doi:10.1073/pnas.0602922103.
21. Jiang, H.; Burdick, D.; Glabe, C.G.; Cotman, C.W.; Tenner, A.J. beta-amyloid activates complement by binding to a specific region of the collagen-like domain of the C1q A chain. *J. Immunol.* **1994**, *152*, 5050-9.
22. Webster, S.; Glabe, C.; Rogers, J. Multivalent binding of complement protein C1q to the amyloid β-Peptide (Aβ) promotes the nucleation phase of aβ aggregation. *Biochem. Biophys. Res. Commun.* **1995**, *217*, 869–875, doi:10.1006/bbrc.1995.2852.

23. Fu, H.; Liu, B.; Frost, J.L.; Hong, S.; Jin, M.; Ostaszewski, B.; Shankar, G.M.; Costantino, I.M.; Carroll, M.C.; Mayadas, T.N.; et al. Complement component C3 and complement receptor type 3 contribute to the phagocytosis and clearance of fibrillar Aβ by microglia. *Glia* **2012**, *60*, 993–1003, doi:10.1002/glia.22331.
24. Shi, Q.; Chowdhury, S.; Ma, R.; Le, K.X.; Hong, S.; Caldarone, B.J.; Stevens, B.; Lemere, C.A. Complement C3 deficiency protects against neurodegeneration in aged plaque-rich APP/PS1 mice. *Sci. Transl. Med.* **2017**, *9*, eaaf6295, doi:10.1126/scitranslmed.aaf6295.
25. Bradt, B.M.; Kolb, W.P.; Cooper, N.R. Complement-dependent proinflammatory properties of the Alzheimer's disease β-Peptide. *J. Exp. Med.* **1998**, *188*, 431–438, doi:10.1084/jem.188.3.431.
26. Trouw, L.A.; Nielsen, H.M.; Minthon, L.; Londos, E.; Landberg, G.; Veerhuis, R.; Janciauskiene, S.; Blom, A.M. C4b-binding protein in Alzheimer's disease: Binding to Aβ1–42 and to dead cells. *Mol. Immunol.* **2008**, *45*, 3649–3660, doi:10.1016/j.molimm.2008.04.025.
27. Milton, N.G.; Harris, J.R. Polymorphism of amyloid-β fibrils and its effects on human erythrocyte catalase binding. *Micron* **2009**, *40*, 800–810, doi:10.1016/j.micron.2009.07.006.
28. Milton, N.G. Amyloid-β binds catalase with high affinity and inhibits hydrogen peroxide breakdown. *Biochem. J.* **1999**, *344*, 293–296, doi:10.1042/0264-6021:3440293.
29. Milton, N.G.N.; Mayor, N.P.; Rawlinson, J. Identification of amyloid-β binding sites using an antisense peptide approach. *NeuroReport* **2001**, *12*, 2561–2566, doi:10.1097/00001756-200108080-00054.
30. Strohmeyer, R.; Ramirez, M.; Cole, G.J.; Mueller, K.; Rogers, J. Association of factor H of the alternative pathway of complement with agrin and complement receptor 3 in the Alzheimer's disease brain. *J. Neuroimmunol.* **2002**, *131*, 135–146, doi:10.1016/s0165-5728(02)00272-2.
31. Wang, J.; Ohno-Matsui, K.; Yoshida, T.; Kojima, A.; Shimada, N.; Nakahama, K.-I.; Safranova, O.; Iwata, N.; Saido, T.C.; Mochizuki, M.; et al. Altered function of factor i caused by amyloid β: implication for pathogenesis of age-related macular degeneration from drusen. *J. Immunol.* **2008**, *181*, 712–720, doi:10.4049/jimmunol.181.1.712.
32. Zlokovic, B.; Martel, C.; Mackic, J.; Matsubara, E.; Wisniewski, T.; McComb, J.; Frangione, B.; Ghiso, J. Brain Uptake of circulating apolipoproteins J and E complexed to Alzheimer's amyloid β. *Biochem. Biophys. Res. Commun.* **1994**, *205*, 1431–1437, doi:10.1006/bbrc.1994.2825.
33. Ghiso, J.; Matsubara, E.; Koudinov, A.; Choi-Miura, N.H.; Tomita, M.; Wisniewski, T.; Frangione, B. The cerebrospinal-fluid soluble form of Alzheimer's amyloid β is complexed to SP-40,40 (apolipoprotein J), an inhibitor of the complement membrane-attack complex. *Biochem. J.* **1993**, *293*, 27–30, doi:10.1042/bj2930027.
34. Yerbury, J.J.; Poon, S.; Meehan, S.; Thompson, B.; Kumita, J.R.; Dobson, C.M.; Wilson, M.R. The extracellular chaperone clusterin influences amyloid formation and toxicity by interacting with prefibrillar structures. *FASEB J.* **2007**, *21*, 2312–2322, doi:10.1096/fj.06-7986com.
35. Beeg, M.; Stravalaci, M.; Romeo, M.; Carrá, A.D.; Cagnotto, A.; Rossi, A.; Diomede, L.; Salmona, M.; Gobbi, M. Clusterin Binds to Aβ1–42 oligomers with high affinity and interferes with peptide aggregation by inhibiting primary and secondary nucleation. *J. Biol. Chem.* **2016**, *291*, 6958–6966, doi:10.1074/jbc.m115.689539.
36. Škerget, K.; Taler-Verčič, A.; Bavdek, A.; Hodnik, V.; Čeru, S.; Tušek-Žnidarič, M.; Kumm, T.; Pitsi, D.; Pompe-Novak, M.; Palumaa, P.; et al. Interaction between oligomers of Stefin B and amyloid-β in vitro and in cells. *J. Biol. Chem.* **2010**, *285*, 3201–3210, doi:10.1074/jbc.m109.024620.
37. Joseph, K.; Shibayama, Y.; Nakazawa, Y.; Peerschke, E.I.; Ghebrehiwet, B.; Kaplan, A.P. Interaction of Factor XII and high molecular weight kininogen with cytokeratin 1 and gC1qR of vascular endothelial cells and with aggregated Aβ protein of Alzheimer's disease. *Immunopharmacology* **1999**, *43*, 203–210, doi:10.1016/s0162-3109(99)00136-8.
38. Antequera, D.; Vargas, T.; Ugalde, C.; Spuch, C.; Molina, J.A.; Ferrer, I.; Bermejo-Pareja, F.; Carro, E. Cytoplasmic gelsolin increases mitochondrial activity and reduces Aβ burden in a mouse model of Alzheimer's disease. *Neurobiol. Dis.* **2009**, *36*, 42–50, doi:10.1016/j.nbd.2009.06.018.
39. Ahn, H.J.; Zamolodchikov, D.; Cortes-Canteli, M.; Norris, E.H.; Glickman, J.F.; Strickland, S. Alzheimer's disease peptide -amyloid interacts with fibrinogen and induces its oligomerization. *Proc. Natl. Acad. Sci. USA* **2010**, *107*, 21812–21817, doi:10.1073/pnas.1010373107.
40. Verdier, Y.; Foldi, I.; Sergeant, N.; Fülöp, L.; Penke, Z.; Janáky, T.; Szücs, M.; Penke, B. Characterization of the interaction between Aβ 1–42 and glyceraldehyde phosphodehydrogenase. *J. Pept. Sci.* **2008**, *14*, 755–762, doi:10.1002/psc.998.
41. Moon, M.; Song, H.; Hong, H.J.; Nam, D.W.; Cha, M.-Y.; Oh, M.S.; Yu, J.; Ryu, H.; Mookjung, I. Vitamin D-binding protein interacts with Aβ and suppresses Aβ-mediated pathology. *Cell Death Differ.* **2013**, *20*, 630–638, doi:10.1038/cdd.2012.161.
42. Chauhan, V.P.; Ray, I.; Chauhan, A.; Wisniewski, H.M. Binding of gelsolin, a secretory protein, to amyloid β-protein. *Biochem. Biophys. Res. Commun.* **1999**, *258*, 241–246, doi:10.1006/bbrc.1999.0623.
43. Ray, I.; Chauhan, A.; Wegiel, J.; Chauhan, V.P. Gelsolin inhibits the fibrillization of amyloid beta-protein, and also defibrillizes its preformed fibrils. *Brain Res.* **2000**, *853*, 344–351, doi:10.1016/s0006-8993(99)02315-x.
44. Yu, Y.; Zhang, L.; Li, C.; Sun, X.; Tang, D.; Shi, G. A Method for evaluating the level of soluble β-amyloid(1-40/1-42)in Alzheimer's disease based on the binding of gelsolin to β-amyloid peptides. *Angew. Chem. Int. Ed.* **2014**, *53*, 12832–12835, doi:10.1002/anie.201405001.

45. Spagnuolo, M.S.; Maresca, B.; La Marca, V.; Carrizzo, A.; Veronesi, C.; Cupidi, C.; Piccoli, T.; Maletta, R.G.; Bruni, A.C.; Abrescia, P.; et al. Haptoglobin interacts with apolipoprotein E and Beta-amyloid and influences their crosstalk. *ACS Chem. Neurosci.* **2014**, *5*, 837–847, doi:10.1021/cn500099f.
46. Yoshiike, Y.; Minai, R.; Matsuo, Y.; Chen, Y.-R.; Kimura, T.; Takashima, A. Amyloid oligomer conformation in a group of natively folded proteins. *PLoS ONE* **2008**, *3*, e3235, doi:10.1371/journal.pone.0003235.
47. Bell, R.D.; Sagare, A.P.; Friedman, A.E.; Bedi, G.S.; Holtzman, D.M.; Deane, R.; Zlokovic, B.V. Transport pathways for clearance of human alzheimer's amyloid β-Peptide and apolipoproteins E and J in the mouse central nervous system. *Br. J. Pharmacol.* **2006**, *27*, 909–918, doi:10.1038/sj.jcbfm.9600419.
48. Wilhelmus, M.M.; Boelens, W.C.; Otte-Holler, I.; Kamps, B.; De Waal, R.M.; Verbeek, M.M. Small heat shock proteins inhibit amyloid-β protein aggregation and cerebrovascular amyloid-β protein toxicity. *Brain Res.* **2006**, *1089*, 67–78, doi:10.1016/j.brainres.2006.03.058.
49. Marcello, A.; Wirths, O.; Schneider-Axmann, T.; Degerman-Gunnarsson, M.; Lannfelt, L.; Bayer, T.A. Circulating immune complexes of Aβ and IgM in plasma of patients with Alzheimer's disease. *J. Neural Transm.* **2009**, *116*, 913–920, doi:10.1007/s00702-009-0224-y.
50. Djogo, N.; Jakovcevski, I.; Müller, C.; Lee, H.J.; Xu, J.-C.; Jakovcevski, M.; Kügler, S.; Loers, G.; Schachner, M. Adhesion molecule L1 binds to amyloid beta and reduces Alzheimer's disease pathology in mice. *Neurobiol. Dis.* **2013**, *56*, 104–115, doi:10.1016/j.nbd.2013.04.014.
51. Ma, Q.; Zhao, Z.; Sagare, A.P.; Wu, Y.; Wang, M.; Owens, N.C.; Verghese, P.B.; Herz, J.; Holtzman, D.M.; Zlokovic, B.V. Blood-brain barrier-associated pericytes internalize and clear aggregated amyloid-β42 by LRP1-dependent apolipoprotein E isoform-specific mechanism. *Mol. Neurodegener.* **2018**, *13*, 1–13, doi:10.1186/s13024-018-0286-0.
52. Sagare, A.; Deane, R.; Bell, R.D.; Johnson, B.; Hamm, K.; Pendu, R.; Marky, A.; Lenting, P.J.; Wu, Z.; Zarcone, T.; et al. Clearance of amyloid-β by circulating lipoprotein receptors. *Nat. Med.* **2007**, *13*, 1029–1031, doi:10.1038/nm1635.
53. Deane, R.; Wu, Z.; Sagare, A.; Davis, J.; Du Yan, S.; Hamm, K.; Xu, F.; Parisi, M.; LaRue, B.; Hu, H.W.; et al. LRP/Amyloid β-peptide interaction mediates differential brain efflux of Aβ isoforms. *Neuron* **2004**, *43*, 333–344, doi:10.1016/j.neuron.2004.07.017.
54. Ozawa, D.; Nakamura, T.; Koike, M.; Hirano, K.; Miki, Y.; Beppu, M. Shuttling Protein Nucleolin Is a microglia receptor for amyloid beta peptide 1-42. *Biol. Pharm. Bull.* **2013**, *36*, 1587–1593, doi:10.1248/bpb.b13-00432.
55. Cater, J.H.; Kumita, J.R.; Abdallah, R.Z.; Zhao, G.; Bernardo-Gancedo, A.; Henry, A.; Winata, W.; Chi, M.; Grenyer, B.S.F.; Townsend, M.L.; et al. Human pregnancy zone protein stabilizes misfolded proteins including preeclampsia- and Alzheimer's-associated amyloid beta peptide. *Proc. Natl. Acad. Sci. USA* **2019**, *116*, 6101–6110, doi:10.1073/pnas.1817298116.
56. Pujadas, L.; Rossi, D.; Andrés, R.; Teixeira, C.M.; Serra-Vidal, B.; Parcerisas, A.; Maldonado, R.; Giralt, E.; Carulla, N.; Soriano, E. Reelin delays amyloid-beta fibril formation and rescues cognitive deficits in a model of Alzheimer's disease. *Nat. Commun.* **2014**, *5*, 3443, doi:10.1038/ncomms4443.
57. Doehner, J.; Madhusudan, A.; Konietzko, U.; Fritschy, J.-M.; Knuesel, I. Co-Localization of reelin and proteolytic AβPP fragments in hippocampal plaques in aged wild-type mice. *J. Alzheimer's Dis.* **2010**, *19*, 1339–1357, doi:10.3233/jad-2010-1333.
58. Hagmeyer, S.; Romão, M.A.; Cristóvão, J.S.; Vilella, A.; Zoli, M.; Gomes, C.M.; Grabrucker, A.M. Distribution and relative abundance of S100 proteins in the brain of the APP23 Alzheimer's disease model mice. *Front. Neurosci.* **2019**, *13*, 640, doi:10.3389/fnins.2019.00640.
59. Baldassarre, M.; Baronio, C.M.; Morozova-Roche, L.A.; Barth, A. Amyloid β-peptides 1–40 and 1–42 form oligomers with mixed β-sheets. *Chem. Sci.* **2017**, *8*, 8247–8254, doi:10.1039/c7sc01743j.
60. Bellinger, F.P.; He, Q.-P.; Bellinger, M.T.; Lin, Y.; Raman, A.V.; White, L.R.; Berry, M.J. Association of Selenoprotein P with Alzheimer's pathology in human cortex. *J. Alzheimer's Dis.* **2008**, *15*, 465–472, doi:10.3233/jad-2008-15313.
61. Strittmatter, W.J.; Saunders, A.M.; Goedert, M.; Weisgraber, K.H.; Dong, L.M.; Jakes, R.; Huang, D.Y.; Pericak-Vance, M.; Schmechel, D.; Roses, A.D. Isoform-specific interactions of apolipoprotein E with microtubule-associated protein tau: Implications for Alzheimer disease. *Proc. Natl. Acad. Sci. USA* **1994**, *91*, 11183–11186, doi:10.1073/pnas.91.23.11183.
62. Du, X.; Wang, Z.; Zheng, Y.; Li, H.; Ni, J.; Liu, Q. Inhibitory effect of selenoprotein P on Cu+/Cu2+-induced Aβ42aggregation and toxicity. *Inorg. Chem.* **2014**, *53*, 1672–1678, doi:10.1021/ic4028282.
63. Giunta, S.; Galeazzi, R.; Marcellini, M.; Corder, E.; Galeazzi, L. The inflammation-sensitive protein alpha 1-anti-chymotrypsin neutralizes fibrillar aggregation and cytotoxicity of the beta-amyloid peptide more effectively than alpha 1-antitrypsin. *Clin. Biochem.* **2007**, *40*, 887–892, doi:10.1016/j.clinbiochem.2007.03.026.
64. Sun, Y.-X.; Wright, H.; Janciauskiene, S. ?1-Antichymotrypsin/Alzheimer's peptide A?1-42 complex perturbs lipid metabolism and activates transcription factors PPAR? and NF?B in human neuroblastoma (Kelly) cells. *J. Neurosci. Res.* **2002**, *67*, 511–522, doi:10.1002/jnr.10144.
65. Raditsis, A.V.; Milojevic, J.; Melacini, G. Aβ association inhibition by transferrin. *Biophys. J.* **2013**, *105*, 473–480, doi:10.1016/j.bpj.2013.03.065.
66. Faye, C.; Chautard, E.; Olsen, B.R.; Ricard-Blum, S. The first draft of the endostatin interaction network. *J. Biol. Chem.* **2009**, *284*, 22041–22047, doi:10.1074/jbc.m109.002964.
67. Du, J.; Cho, P.Y.; Yang, D.T.; Murphy, R.M. Identification of beta-amyloid-binding sites on transthyretin. *Protein Eng. Des. Sel.* **2012**, *25*, 337–345, doi:10.1093/protein/gzs026.

68. Schwarzman, A.L.; Gregori, L.; Vitek, M.P.; Lyubski, S.; Strittmatter, W.J.; Enghilde, J.J.; Bhasin, R.; Silverman, J.; Weisgraber, K.H.; Coyle, P.K. Transthyretin sequesters amyloid beta protein and prevents amyloid formation. *Proc. Natl. Acad. Sci. USA* **1994**, *91*, 8368–8372, doi:10.1073/pnas.91.18.8368.
69. Rosa, I.M.; Henriques, A.G.; Carvalho, L.; Oliveira, J.; Silva, O.A.D.C.E. Screening younger individuals in a primary care setting flags putative dementia cases and correlates gastrointestinal diseases with poor cognitive performance. *Dement. Geriatr. Cogn. Disord.* **2016**, *43*, 15–28, doi:10.1159/000452485.
70. Rosa, I.M.; Henriques, A.G.; Wiltfang, J.; Silva, O.A.D.C.E. Putative dementia cases fluctuate as a function of mini-mental state examination cut-off points. *J. Alzheimer's Dis.* **2017**, *61*, 157–167, doi:10.3233/jad-170501.
71. Saman, S.; Kim, W.; Raya, M.; Visnick, Y.; Miro, S.; Saman, S.; Jackson, B.; McKee, A.C.; Alvarez, V.E.; Lee, N.C.; et al. Exosome-associated Tau is secreted in tauopathy models and is selectively phosphorylated in cerebrospinal fluid in early Alzheimer disease. *J. Biol. Chem.* **2012**, *287*, 3842–3849, doi:10.1074/jbc.m111.277061.
72. Agliardi, C.; Guerini, F.R.; Zanzottera, M.; Bianchi, A.; Nemni, R.; Clerici, M. SNAP-25 in serum is carried by exosomes of neuronal origin and is a potential biomarker of Alzheimer's disease. *Mol. Neurobiol.* **2019**, *56*, 5792–5798, doi:10.1007/s12035-019-1501-x.
73. Goetzl, E.J.; Mustapic, M.; Kapogiannis, D.; Eitan, E.; Lobach, I.V.; Goetzl, L.; Schwartz, J.B.; Miller, B.L. Cargo proteins of plasma astrocyte-derived exosomes in Alzheimer's disease. *FASEB J.* **2016**, *30*, 3853–3859, doi:10.1096/fj.201600756r.
74. Goetzl, E.J.; Kapogiannis, D.; Schwartz, J.B.; Lobach, I.V.; Goetzl, L.; Abner, E.L.; Jicha, G.A.; Karydas, A.M.; Boxer, A.; Miller, B.L. Decreased synaptic proteins in neuronal exosomes of frontotemporal dementia and Alzheimer's disease. *FASEB J.* **2016**, *30*, 4141–4148, doi:10.1096/fj.201600816r.
75. Winston, C.N.; Goetzl, E.J.; Schwartz, J.B.; Elahi, F.M.; Rissman, R.A. Complement protein levels in plasma astrocyte-derived exosomes are abnormal in conversion from mild cognitive impairment to Alzheimer's disease dementia. *Alzheimer's Dement. Diagn. Assess. Dis. Monit.* **2019**, *11*, 61–66, doi:10.1016/j.dadm.2018.11.002.
76. Winston, C.N.; Goetzl, E.J.; Akers, J.C.; Carter, B.S.; Rockenstein, E.M.; Galasko, D.; Masliah, E.; Rissman, R.A. Prediction of conversion from mild cognitive impairment to dementia with neuronally derived blood exosome protein profile. *Alzheimer's Dementia: Diagn. Assess. Dis. Monit.* **2016**, *3*, 63–72, doi:10.1016/j.dadm.2016.04.001.
77. Björkdahl, C.; Sjögren, M.J.; Zhou, X.; Concha, H.; Avila, J.; Winblad, B.; Pei, J.-J. Small heat shock proteins Hsp27 or αB-crystallin and the protein components of neurofibrillary tangles: Tau and neurofilaments. *J. Neurosci. Res.* **2007**, *86*, 1343–1352, doi:10.1002/jnr.21589.
78. Goetzl, E.J.; Boxer, A.; Schwartz, J.B.; Abner, E.L.; Petersen, R.C.; Miller, B.L.; Carlson, O.D.; Mustapic, M.; Kapogiannis, D. Low neural exosomal levels of cellular survival factors in Alzheimer's disease. *Ann. Clin. Transl. Neurol.* **2015**, *2*, 769–773, doi:10.1002/acn3.211.
